# Cavity Controlled Upconversion in CdSe Nanoplatelet Polaritons

**DOI:** 10.1021/acsnano.4c05871

**Published:** 2024-07-30

**Authors:** Mitesh Amin, Eric R. Koessler, Ovishek Morshed, Farwa Awan, Nicole M. B. Cogan, Robert Collison, Trevor M. Tumiel, William Girten, Christopher Leiter, A. Nickolas Vamivakas, Pengfei Huo, Todd D. Krauss

**Affiliations:** † The Institute of Optics, University of Rochester, Rochester, New York 14627, United States; ‡ Department of Chemistry, University of Rochester, Rochester, New York 14627, United States; ¶ Department of Chemistry, Regis University, Denver, Colorado 80221, United States; ∥ Department of Physics and Astronomy, University of Rochester, Rochester, New York 14627, United States

**Keywords:** polariton chemistry, strong coupling, quantum dynamics, CdSe nanoplatelets, upconversion

## Abstract

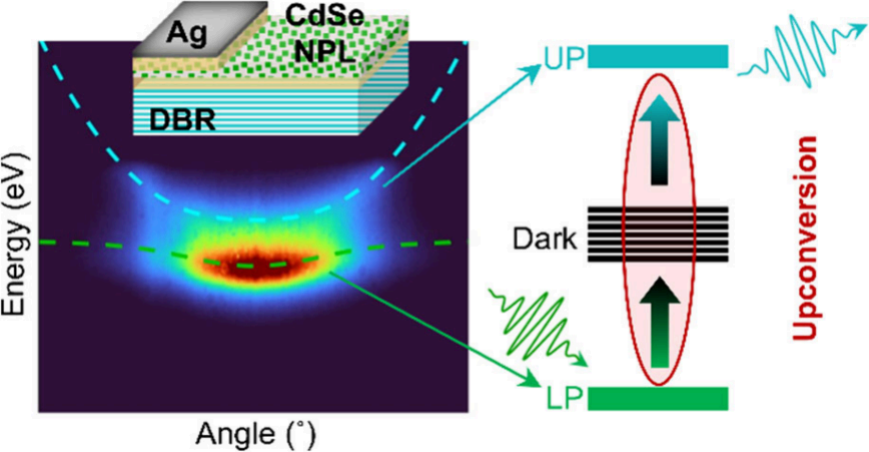

Exciton-polaritons provide a versatile platform for investigating quantum electrodynamics effects in chemical systems, such as polariton-altered chemical reactivity. However, using polaritons in chemical contexts will require a better understanding of their photophysical properties under ambient conditions, where chemistry is typically performed. Here, we used cavity quality factor to control strong light–matter interactions and in particular the excited state dynamics of colloidal CdSe nanoplatelets (NPLs) coupled to a Fabry–Pérot optical cavity. With increasing cavity quality factor, we observe significant population of the upper polariton (UP) state, exemplified by the rare observation of substantial UP photoluminescence (PL). Excitation of the lower polariton (LP) states results in upconverted PL emission from the UP branch due to efficient exchange of population between the LP, UP and the reservoir of dark states present in collectively coupled polaritonic systems. In addition, we measure time scales for polariton dynamics ∼100 ps, implying great potential for NPL based polariton systems to affect photochemical reaction rates. State-of-the-art quantum dynamical simulations show outstanding quantitative agreement with experiments, and thus provide important insight into polariton photophysical dynamics of collectively coupled nanocrystal-based systems. These findings represent a significant step toward the development of practical polariton photochemistry platforms.

The quantum-mechanical coupling of the electronic states of matter to the electromagnetic modes of an optical cavity results in formation of two hybrid light–matter eigenstates known as the upper polariton (UP) and lower polariton (LP). For nanomolecular systems (such as colloidal nanocrystals^1,2^) that have an excitonic photoexcited state, these light–matter eigenstates are termed exciton-polaritons. Recent advances in the field of exciton-polariton systems have led to their proposed use for altering chemical reactivity,^3−11^ enhancing intermolecular energy transfer,^12−15^ enabling room temperature Bose–Einstein condensates,^16,17^ and providing qubits for quantum simulation.^18,19^ With respect to the specific application of using polaritons for altering chemical reactivity, polaritons are thought to alter frontier molecular orbitals as well as orbital energetics. For example, calculations suggest that strong light–molecule coupling can dramatically increase charge transfer rates by a few orders of magnitude, alter electron–phonon coupling, and modify energy landscapes.^20−24^ Indeed, recent experimental work has shown that strong coupling to the electronic states of photoswitchable molecules can modify the kinetics of their isomerization.^10^ Strong coupling to the vibrational modes of molecules has led to changes in ground state chemical reactivity, including modifying the distribution of products from a disassociation^25,26^ and the suppression of reaction rates for alcoholysis of phenyl isocyanate with cyclohexanol.^11^

While polariton photochemistry provides exciting promise for altering chemical transformations, fulfilling that promise will require a thorough understanding of how optical cavities can be used to control polariton photophysics, which is an active area of research. For instance, molecular polaritonic systems often operate in the collective coupling regime, whereby the polariton state is a single, coherent, quantum-mechanical superposition of excitations from thousands to millions of molecules.^27^ However, this collective coupling also results in a dense manifold of optically inactive exciton states that only weakly couple with the cavity photon.^22,28^ Having mostly matter (i.e., excitonic) character, this exciton reservoir of dark states dominates the LP and UP dynamics,^29−32^ leading to the question of whether modified chemical reactivity is even possible under these collective coupling conditions.^33,34^ Alternatively, for colloidal nanocrystal based polariton systems, which have orders of magnitude fewer emitters coupled to the cavity,^35^ the influence of the dark state reservoir on the photophysics of the UP and LP is largely unexplored, and thus could provide distinct opportunities for nanomolecular polariton systems.^36,37^

In this work, we explore tuning the exciton-polariton photophysics of 2D cadmium selenide (CdSe) nanoplatelets (NPLs) strongly coupled to low and high quality (Q)-factor Fabry–Pérot (FP) optical cavities. By reducing the cavity loss rate, we observed photoluminescence (PL) from the UP state due to efficient population transfer from the reservoir of dark states, as verified by both experiments and quantum dynamical calculations. In fact, in the high-quality cavity, the relatively strong coupling between the UP, LP and dark states allowed for photoexcitation at the LP energy, which normally would not be absorbed by the NPLs, to be upconverted to create a finite population in the UP. The polariton dynamics of these higher-Q cavities under ambient conditions led to measured polariton PL lifetimes on the order of 100 ps, which are long enough to provide a fundamental basis for NPL polariton systems to affect photochemical reaction rates.

## Results and Discussion

### Polariton Dispersion Characteristics

**Figure 1:**
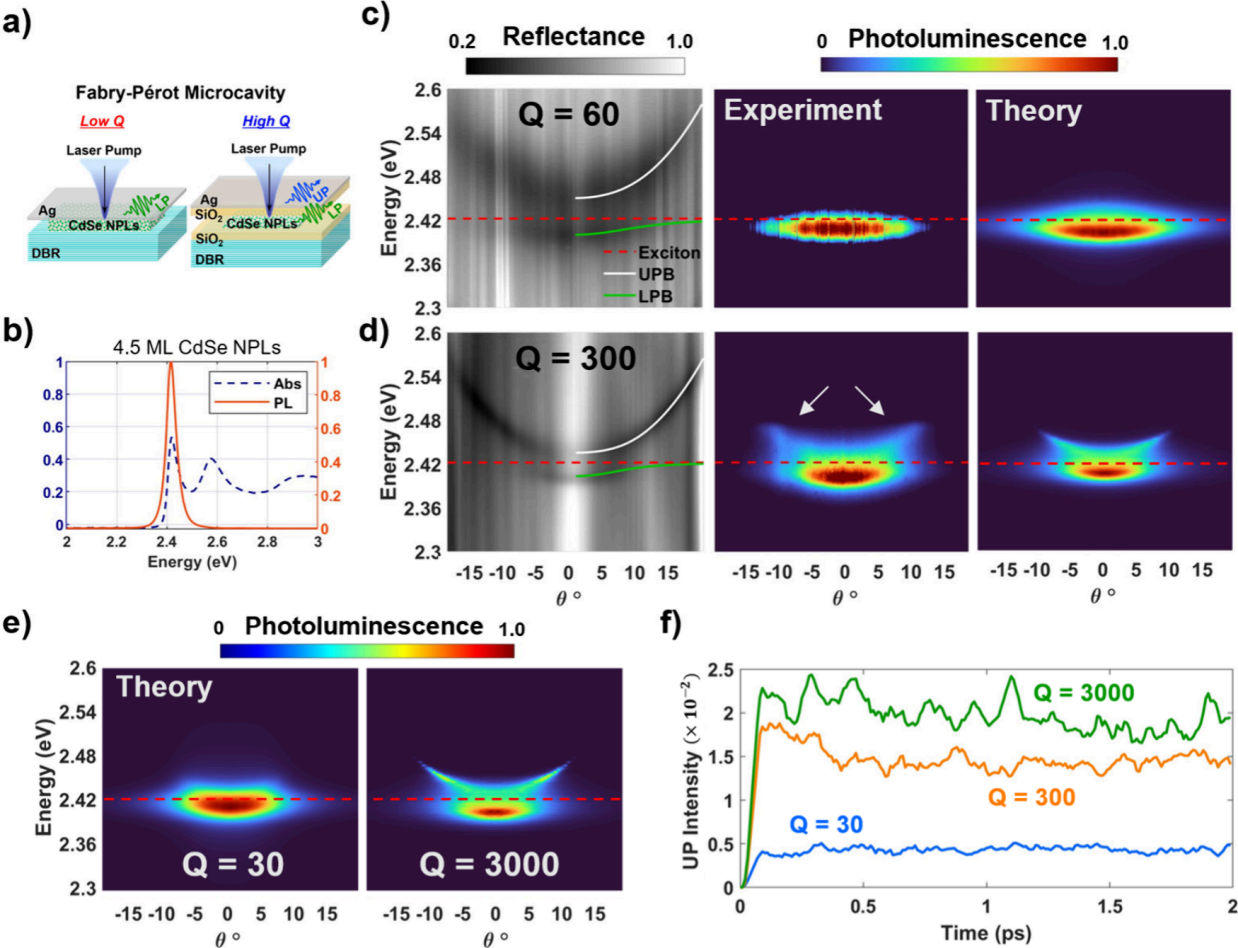
Strong coupling of CdSe NPLs in a microcavity at room temperature. (a) Metal-dielectric DBR cavity geometry for a lower cavity quality factor *Q* = 60 and for a higher *Q* = 300 with SiO_2_ spacers. (b) Absorbance (dotted blue) and PL (red) of 4.5 ML CdSe NPLs in solution. (c) Angle-resolved reflectance (left) and PL spectra (experimental middle, simulated right) with fitted UPB (white), LPB (green), and exciton heavy-hole transition energy at 2.42 eV (dotted red) overlay for a sample corresponding to detuning energy Δ= +6 *meV*, Rabi-splitting energy *ℏ*Ω_*R*_ = 50 *meV*, and *Q* = 60. (d) Same as (c) but with Δ = −1 *meV*, *ℏ*Ω_*R*_ = 31 *meV*, *Q* = 300. Corresponding simulated quantum dynamical simulations showing excellent agreement with measured spectra with white arrows indicating UPB PL emission. (e) Simulated ARPL spectra for varying Q-factor or cavity loss rates for experimental conditions in (d). (f) Theoretical calculations of the UP populations above 2.437 eV weighted by photonic character (which is proportional to emission intensity), indicating greater UPB population buildup for increasing cavity Q-factor.

CdSe NPLs have been recently explored in various exciton-polaritonic systems^38−42^ due to their well-defined narrow absorption and photoluminescence (PL) line widths (∼40 meV), high oscillator strengths, and small Stokes shifts (∼5–10 meV), making them excellent materials for achieving and investigating strong light–matter coupling.^43−46^ Here, we integrated 4.5 monolayer CdSe NPLs (approximate lateral size of 22 nm × 15 nm, see Supporting Information (SI) for fabrication details) into two types of FP microcavities with varying cavity Q-factors as illustrated in Figure 1a. For FP cavities, the cavity frequency ω_**k**_ depends on the wavevector of the mode and can be expressed as
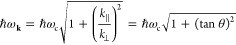
where  is the cavity energy at normal incidence, *c* is the speed of light, *n* is the refractive index inside the cavity, *k*_∥_ and *k*_⊥_ are the wavevector components of the photon mode which are parallel and perpendicular to the cavity mirrors, respectively, and  is the angle of incidence and emission. The perpendicular wavevector component *k*_⊥_ is fixed and only the parallel component *k*_∥_ will vary as a function of emission angle θ, thus giving rise to the angular dependence (i.e., dispersion) of the cavity mode (Figure S3). The Q-factor of a cavity is , where γ_c_ is the bare cavity loss rate. For the high-Q factor cavity, the heavy hole (HH) absorption transition defined at 2.42 eV (Figure 1b) is in resonance with the fundamental mode of the 3λ/2*n* cavity, where a thin 60 nm NPL film is deposited at the antinode of the cavity having an effective index of refraction *n*. For the lower Q cavity, a thicker 110 nm NPL layer is deposited in resonance with the first order λ/2*n* mode cavity.

We hypothesized that increasing the Q-factor of the FP cavity would enable different polariton photophysical behavior, due to the significant effect of cavity loss on polariton population dynamics.^47,48^ Thus, our low Q-factor cavity serves as a control experiment and an important benchmark for our quantum dynamics simulations to accurately describe any emergent behavior associated with varying cavity loss for the CdSe NPL polariton systems. As illustrated in Figure 1a, compared to a cavity that is entirely filled with NPLs,^49^ we calculated that adding inert spacer layers between the NPLs and the mirrors would greatly increase the FP cavity Q-factor. Indeed, adding two 200 nm SiO_2_ spacer layers between the NPL layer and the two mirror surfaces provided a Q-factor of approximately 300, compared to a Q-factor of about 70 without the spacers (Figure S1). A 31-layer distributed Bragg reflector (DBR) consisting of alternating layers of Si_3_N_4_ and SiO_2_ are used to form the bottom 99.9% reflective mirror in the 2.25–2.75 eV band, while 40 nm of silver deposited on the top spacer layer acts as the top mirror to provide sufficient in and out coupling of light to form the full cavity.

An angle-resolved (Fourier-space) spectroscopy system with a 40X/0.6 NA microscope objective corresponding to a ±19°collection range (defined by angle inside the active cavity layer) was implemented to measure the cavity angle-resolved reflectance (ARR) and angle-resolved PL (ARPL) spectra. Figure 1c-d shows the ARR spectra for the two cavities with the NPL HH exciton in close resonance with the cavity near zero angle. For the higher Q cavity in Figure 1d, we see the clear emergence of the two UP and LP absorbing branches split about the uncoupled exciton energy. Both branches of the ARR spectra are fitted with a coupled harmonic oscillator model to extract the Rabi splitting energy in the range of 31–45 meV across four detuning energies (Figure S3). The detuning energy, Δ, is defined by the energy difference between the cavity resonance at zero angle and the NPL exciton heavy hole transition at 2.42 eV. For the lower Q cavity in Figure 1c, we observe much broader UP and LP absorbing branches and obtain slightly higher Rabi splitting energies in the range of 50–70 meV due to a thicker layer of NPLs.

Corresponding ARPL spectra for all detuning values are taken along various sample positions with 2.56 eV laser excitation. For highly negatively detuned cases (cavity energy less than PL exciton energy, Figure S3), the PL intensity is exclusively on the LP branch at the angle that corresponds to the NPL-cavity resonance condition. The lack of PL from the UP and the concentration of PL at a small range of collection angles is in agreement with previous NPL-FP polariton systems,^35^ as well as what has been reported for other exciton-polariton systems.^38,50,51^ However, for the higher Q cavities, as the detuning approaches zero and transitions positive, we observed significant PL emission from the UP branch (in addition to the LP branch) as indicated by the white arrows in Figure 1d (see also Figure S4). For the blue detuned (Δ = +35 meV) cavity in Figure S4, a continuum of PL emission can be seen in the ARPL spectra near zero incident angle toward the UP branch. This is in contrast with the lower Q cavity in Figure 1c where only PL emission from the LP branch is observed. PL emission from the UP branch is exceptionally rare at room temperature, having been reported for only a few polaritonic systems involving organic semiconductors (i.e., *J-aggregated* dyes).^52−55^ While UP PL has been observed for very high-Q DBR-DBR cavities involving 2D inorganic epitaxial III–V quantum well systems,^56−58^ their typical operation at cryogenic temperatures along with stringent device fabrication and lower Rabi splitting energies make them less practical than solution-processed colloidal systems for any potential polariton chemistry platforms. This rare observation of UP PL in nanomolecular systems is, in part, because the fundamental cavity and exciton properties that affect the full dynamics of the UP, LP and dark state populations, are not well understood.

### Physical Origin of Upper Polariton Emission

To better understand the NPL and cavity characteristics that dictate the UP and LP populations as indicated by the measured ARPL spectra, we performed mixed quantum-classical dynamics simulations of the combined NPL-cavity system to calculate the polariton population dynamics and the corresponding PL spectra intensity. The dynamics were propagated using the Lindblad-MASH (L-MASH) method that combines the multistate mapping approach to surface hopping (MASH) method^59,60^ with Lindblad dynamics^48^ to account for cavity loss. The NPL-cavity system was modeled using the generalized Holstein–Tavis–Cummings (GHTC) model, which has been previously used to study light–matter hybrid systems in FP cavities.^22,61^ The HTC Hamiltonian includes several phonon-coupled matter excitations coupled to several angle-dependent cavity photon modes, which allows for the calculation of angle-resolved properties of polariton systems including the effect of the reservoir of dark states. The GHTC Hamiltonian can be expressed as
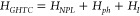
where *H*_*NPL*_ describes the exciton states of *N* independent nanoplatelets, *H*_*ph*_ is the Hamiltonian for the quantized cavity mode, and *H*_*I*_ describes the matter-cavity interactions (between *H*_*NPL*_ and *H*_*ph*_). The full details of this model can be found in the SI Appendix.

To calculate the angle-resolved PL, the population dynamics of the GHTC model with cavity loss were propagated assuming an incoherent driving of population from an initially populated ground state to the HH states. The steady-state populations arising from this propagation were used to calculate the intensities of the PL spectra shown in Figure 1c-d by weighting the angle-dependent photonic character of the polariton states by their steady-state populations.^61^ For both cavities in Figure 1c-d, the calculated PL spectra show excellent agreement with experimental spectra in both the shape of the PL dispersion and the distribution of intensity as a function of PL energy. In particular for the lower Q = 60 cavity, the calculated PL correctly predicts the emission exclusively from the LP branch near zero angle. For the Q = 300 cavity, the simulations also accurately predict the LP dispersion along with the PL intensity on the UP branch up to around 2.47 eV. For the positive detuning case (Δ = +35 meV) cavity in Figure S4, the calculated PL also shows good agreement with the measured PL dispersion. These results indicate that the PL spectra obtained from the L-MASH method with the GHTC model can predict experimental PL spectra of these NPL-FP systems with near quantitative accuracy, demonstrating L-MASH as a powerful state-of-the-art simulation tool for such complex polariton systems with cavity loss and many vibrationally coupled molecules.

The presence of UP PL emission in the simulations can be understood from the perspective of population transfer among a manifold of polariton and dark states. In the simplified picture of strong light–matter coupling (i.e., the Tavis-Cummings (TC) model^22^) the optically bright polariton states are completely orthogonal to the large reservoir of dark exciton states,^52^ and thus they do not exchange excited population. However, more sophisticated treatments (i.e., GHTC Hamiltonian) include the influence of phonons coupling to the dark states, which causes them to become energetically disordered and to gain some photonic character (rendering them only quasi-dark). The net result is that the NPL phonons cause a nonadiabatic coupling between the polariton and the quasi-dark states which leads to population transfer among these states over time.

### Tuning of Upper Polariton Emission with Q-Factor

We hypothesized that the observance of PL from the UP was primarily due to the higher Q-factor optical cavity, which has less photon loss than is normally found in other polariton systems,^23,62^ thereby allowing for a greater chance of populating states with larger photonic character (*i.e*., the higher-angle UP states) before the photon exits the cavity. To investigate this possibility, we simulated polariton PL spectra with different Q-factors (Figure 1e) for the fixed experimental conditions in Figure 1d to understand the effect of cavity loss on polariton dynamics. For small Q-factors (Q = 30), the PL intensity is primarily in the LP branch, in agreement with previous measurements of polariton PL for nanomolecular exciton-polaritons involving organics,^62−64^ carbon nanotubes,^65,66^ and previous CdSe NPL cavity systems.^35,49^ In contrast, for large Q-factors (Q = 3000) there is even greater PL intensity from the UP relative to the (Q = 300) measurements and simulation. This trend is also observed in the dynamics of the excited UP populations defined for energies above 2.437 eV weighted by photonic character (which are proportional to the emission intensity)^67^ in the simulation (Figure 1f) where the higher Q simulations have larger weighted steady-state populations of UP states.

Our simulations confirm the hypothesis that lower cavity loss promotes UP emission and allow for further insight into the distinct polariton dynamics for high Q-factor optical cavities. In particular, for the higher angles of the UP branch to become populated from the quasi-dark state reservoir, the population must first traverse along a manifold of states with various mixtures of photonic character (which straddle the definition between optically “dark” and “bright” states) (Figure S7). The traversing population can only reach the photonic UP branch (i.e., successfully upconvert from the dark state reservoir to the UP) if the cavity loss rate experienced by the partially photonic manifold of states is sufficiently small. Thus, the amount of PL emission from states with significant photonic character on the UP should strongly depend on the Q-factor of the cavity.

**Figure 2:**
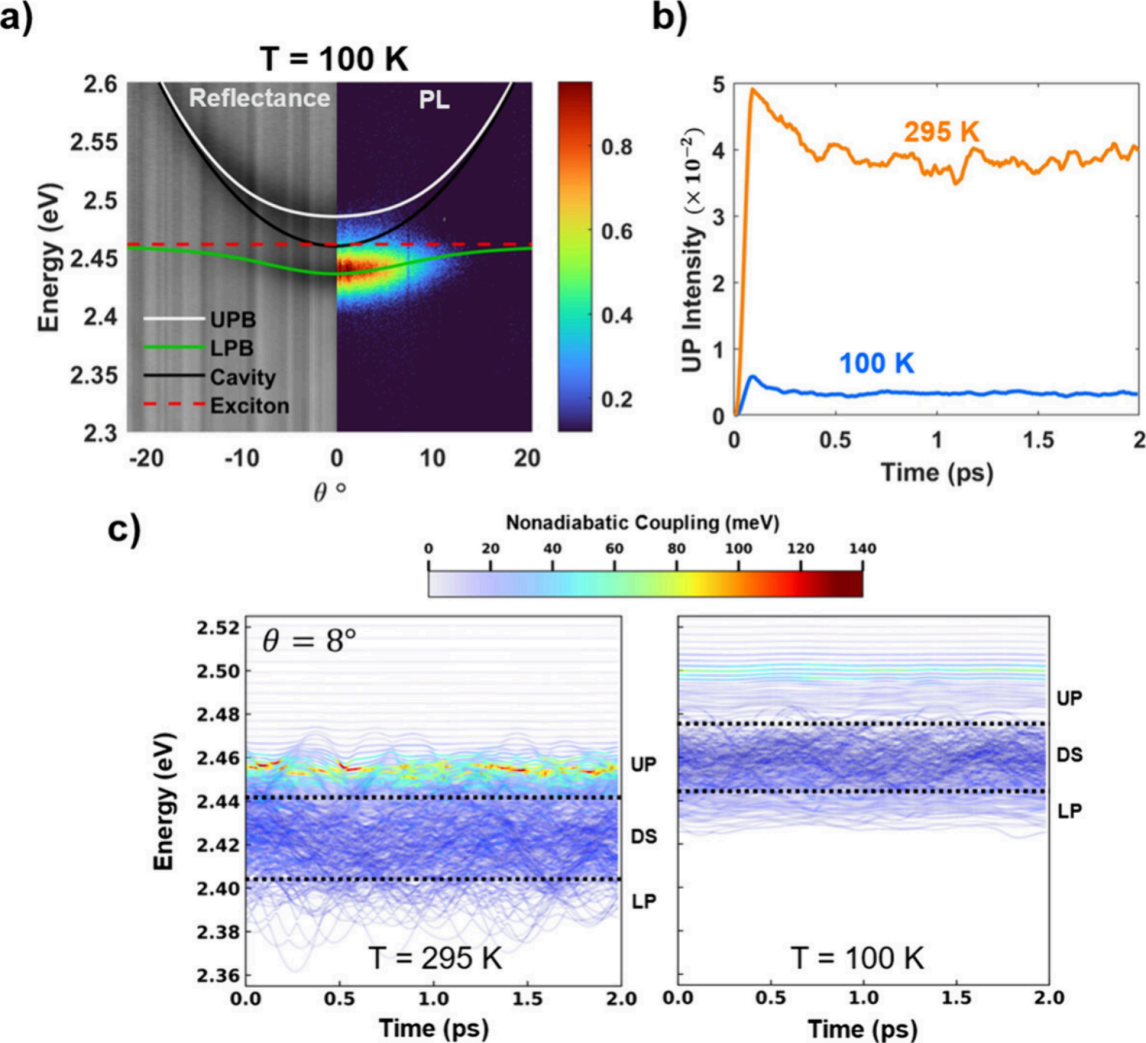
Upper polariton state populations at 295 K vs 100 K. (a) Angle-resolved reflectance and PL spectra taken at 100 K for near resonant detuning of Δ = −1 meV, *ℏ*Ω_*R*_ = 49 meV with fitted polariton branches showing suppressed UP PL emission. (b) Theoretical calculations of the UP populations above the HH exciton energy weighted by photonic character, indicating greater UPB emission at 295 K versus 100 K. (c) Polariton state energies through time of an arbitrary trajectory colored by the magnitude of nonadiabatic coupling due to exchange of character of the cavity mode near 8 degrees, indicating larger UP population transfer rates at 295 K versus 100 K (see SI for full nonadiabatic coupling expression). In the simulation, the orange and red regions of the plot correspond to larger coupling values, which promote population transfer from the dark states (DS) to the UP.

Since phonons drive the upconversion of excited population from the dark states to the UP, we would expect a drastic reduction of the upconverted PL intensity from the UP branch at low temperatures. To that end, ARR and ARPL spectra along two sample positions with similar detuned cavity energies (Δ ∼ −1 meV) were collected and compared at 295 and 100 K (Figure 2a). Two different cavity sample positions were chosen for comparison to account for the temperature dependent HH exciton blue-shift from 2.42 to 2.46 eV (Figure S8).^68^ While CdSe NPLs are reported to have dominant trion PL emission ∼30 meV red-shifted from the HH exciton at cryogenic temperatures,^69,70^ our thin film reflectance measurements at 100 K indicate only blue-shifted HH and LH transitions, with an absence of direct trion absorption due to its small oscillator strength. In addition to the lack of a middle polariton branch which should arise under strong coupling of both the exciton and trion states,^71,72^ we determine our cavities at 100 K only couple to the HH exciton transition. Inside the cavity at 100 K, PL emission is observed solely from the LP branch for small incident angles (±7°), in direct contrast to the enhanced UP PL emission out to higher angles (±12°) for measurements taken at 295 K.

This reduction in UP emission at lower temperatures was supported by simulations of polariton population dynamics performed at 100 and 295 K. Indeed, the weighted populations of the UP states (Figure 2b) were roughly 10-fold smaller in the 100 K simulation versus the simulation at 295 K. This significant difference in UP populations can be explained by the difference in nonadiabatic coupling magnitude between the polariton states at different temperatures. To visualize this difference, in Figure 2c we plot the energies of the polariton states through time for a cavity mode near 8 degrees off vertical. The different colors in Figure 2c show the magnitude of nonadiabatic coupling that each polariton state experiences due to the exchange of character between exciton and photon. Since most of the photonic character of this 8 degree cavity mode is shared among UP polariton states, larger values of coupling indicate faster population transfer to these UP states. The UP states in the 295 K simulation experience over twice the amount of nonadiabatic coupling magnitude as those in the 100 K simulation thus the population is transferred more quickly in the UP at room temperature. This faster population transfer rate allows for population to move further uphill along the UP branch at 295 K before cavity loss can significantly deplete it, while at 100 K, the cavity loss depletes the UP population before it can move further uphill along the UP branch.

### Polariton Upconversion Enabled by High Q-factor Cavity

**Figure 3:**
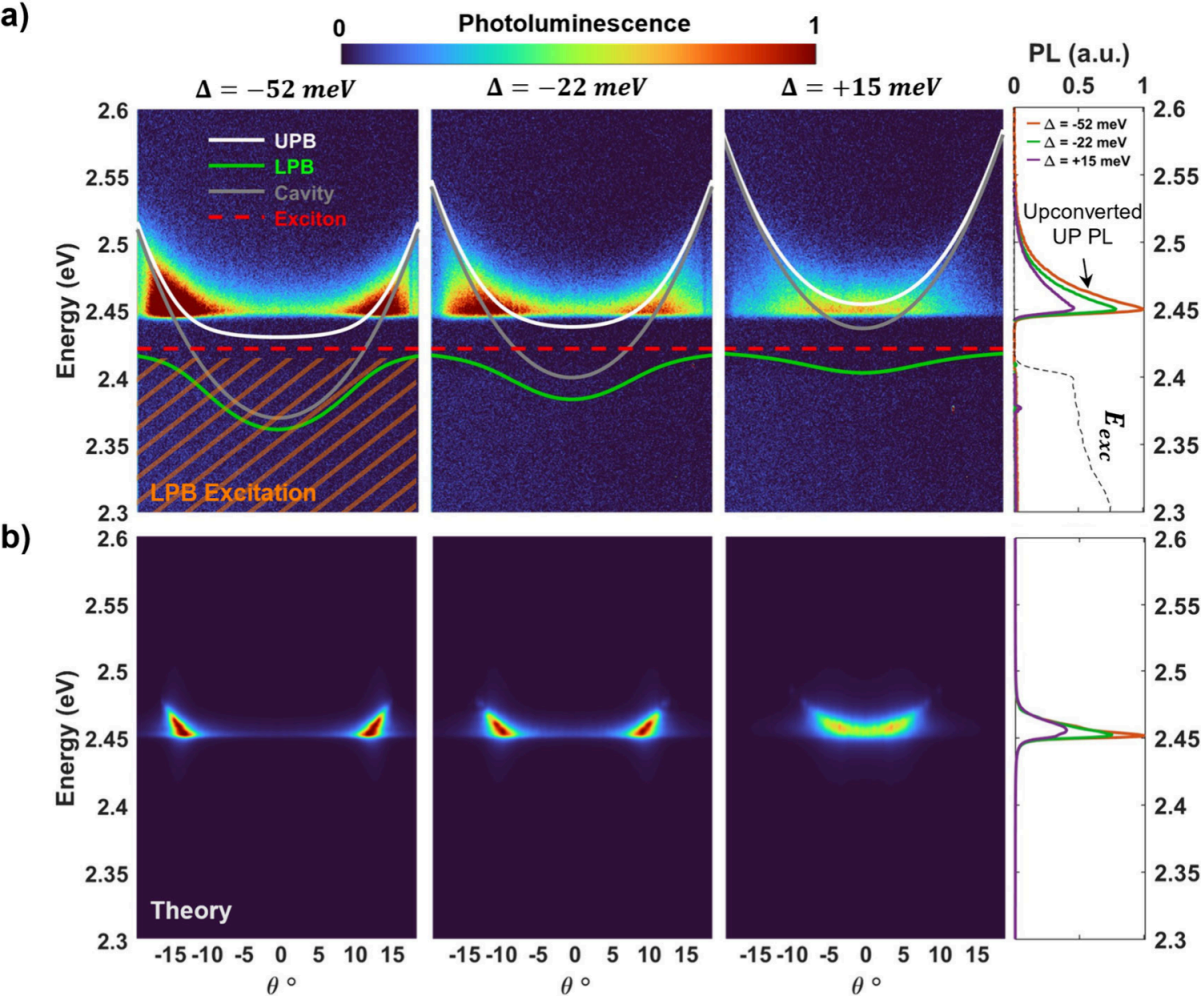
PL upconversion and emission from the upper polariton branch. (a) Angle-resolved PL spectra from a cavity sample with −52, −22, and +15 meV detuning and 45–49 meV Rabi splitting energies showing UPB (white) emission (2.44–2.52 eV) near the cavity-exciton resonance with LPB excitation (2.3–2.41 eV). The LPB is indicated by the green line and the HH exciton energy (2.42 eV) is indicated by the red-dotted line. Integrated linecuts of PL spectra showing enhanced upconverted PL for higher detuned sample positions resulting from more accessible lower-polariton states across the wider in-plane momenta range. (b) Theoretical simulations of PL spectra in (a). Polariton states of energy 2.4 eV and below were pumped with rates weighted by photonic character. Steady-state populations of polariton states of energy 2.45 eV and above were used to calculate PL spectra. Angular dependence of the ARPL and relative intensities across the detunings of the integrated linecuts of PL closely match the measured results in (a).

A central premise motivating the study of polaritons for altering chemical transformations is that the polariton state has fundamentally different physical properties compared to the purely electronic states of matter.^22^ For example, predictions that strong light–matter coupling can be leveraged to enable charge transfer to energetically forbidden molecular acceptors rely on relatively efficient population transfer between the LP, UP and the dark states.^21^ To test the limits of polaritonic population transfer in our system, we resonantly excited the LP branch (2.3–2.41 eV) under the same laser fluence for three different cavity detuned sample positions (Figure 3) having similar Rabi splitting energies in the range of 45–49 meV.

In the absence of strong coupling between the HH NPL exciton and the cavity, light at these wavelengths does not have enough energy to span the NPL bandgap, and thus is not expected to be absorbed significantly. However, upon excitation of the LP branch in all three cases, we observed PL upconversion to the UP branch as shown in Figure 3a. This emergence of UP emission upon LP excitation clearly shows the effectiveness of population transfer from the LP to UP via phonon-mediated interactions with the dark states.

Angle-integrated linecuts along the ARPL spectra reveal that the upconverted PL intensity is highest for the most negatively detuned cavity with a decreasing near-linear dependence for more positive detuning. We attribute this intensity decrease to fewer available LP states in the pumping region as the cavity is increasingly positively detuned, reducing the total amount of absorbed excitation light. This is supported by theoretical simulations of the upconverted PL where LP states were pumped and steady-state UP PL spectra were calculated in Figure 3b. The relative peak intensities of the simulated PL across the three detunings were a near exact match to the measured peak intensities. These simulations, which differ only by their detuning, suggest that the upconverted UP population is originating not only from pumped LP states closest in energy to the UP but also from states along the entire LPB dispersion. This means that upconversion can efficiently occur from LP to UP states with energy gaps as large as 80 meV due to the presence of nonadiabatic coupling across the near continuum of polariton states spanning these energy gaps. As a control, a half cavity thin film NPL sample was excited using 1% of the incident power excitation compared to the cavity samples to account for the 99% cavity reflectivity (and the lower outcoupling UP PL efficiency) in the same 2.3–2.41 eV range. While some weak anti-Stokes PL from the half-cavity is observed (Figure S10) due to small absorption of the homogeneously broadened HH transition, this PL did not have angular dependence and did not extend as high in energy as the PL from the cavity samples due to a lack of UP states at those higher energies. This confirms that the upconverted PL in the cavity samples was primarily emitted from UP states instead of uncoupled excitonic states.

### Polariton Photoluminescence Lifetimes

The simplest picture characterizes the polariton lifetime as a superposition of the radiative rates of the matter and photonic characters of the polariton weighted by the Hopfield coefficients.^73^ Thus, the polariton has a lifetime bounded by the photon lifetime in the cavity (lower bound) and the uncoupled NPL exciton lifetime (upper bound). However, this idealized picture does not account for polariton coupling to the dark states reservoir, leading to measured lifetimes in collective-coupled exciton-polariton systems near equal to or even longer than the uncoupled exciton lifetime.^31,73−76^

**Figure 4:**
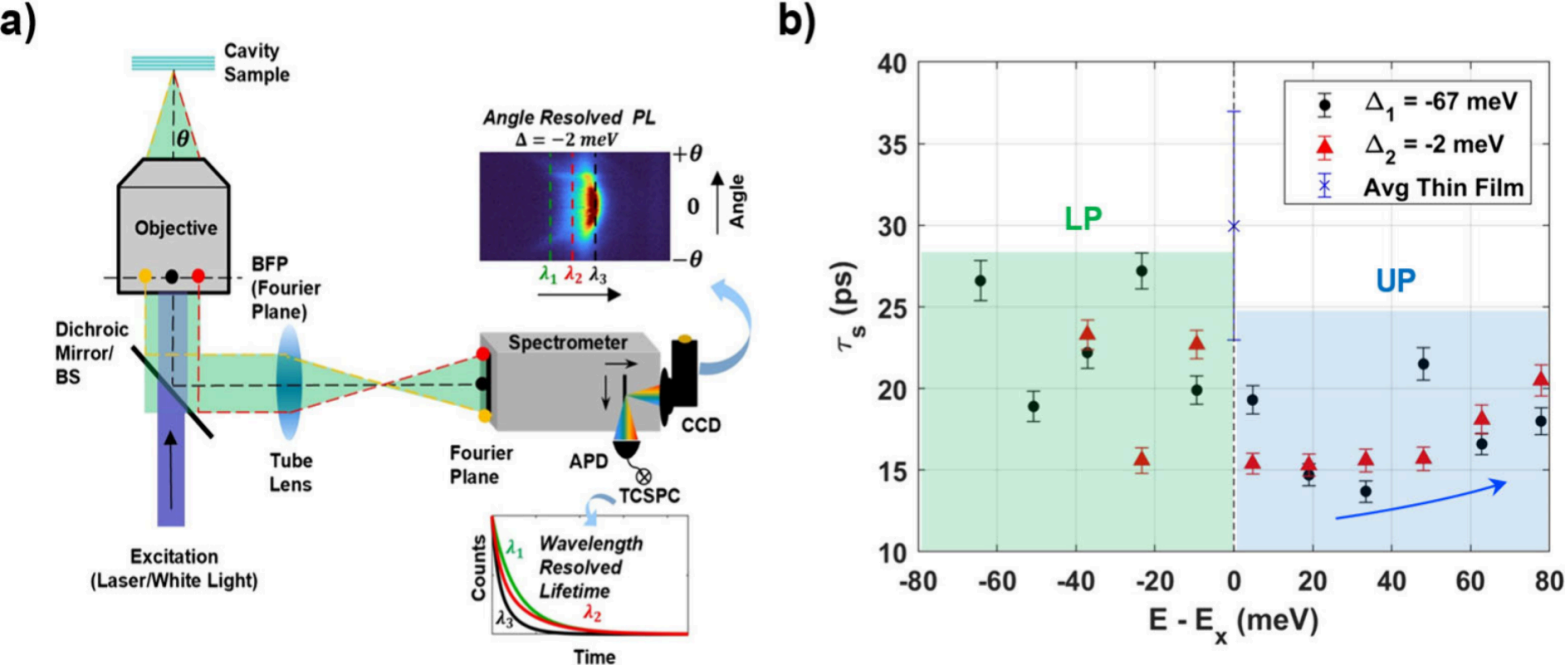
Energy resolved lifetime measurements of the LPB and UPB. (a) Experimental schematic showing correlated angle resolved spectroscopy with high resolution TCSPC via a dual exit port spectrometer. Lifetime measurements are taken along every 3 nm of the emission window as indicated with dotted lines in PL spectra inset. (b) Fitted lifetime values for the short component plotted against the energy difference between the bare exciton emission and the polariton emission energy for two different cavity detuning Δ_1_ = −67 meV, Δ_2_ = −2 meV. Short component lifetime at peak exciton emission for a bare half-cavity thin film sample averaged along three different positions indicated by the blue ‘x’ marker.

To better understand polariton lifetimes from the NPL-FP cavity system with a large Q-factor, we performed high temporal and wavelength resolved time correlated single photon counting (TCSPC) measurements with a dual exit port spectrometer as shown in Figure 4a. By using the ARR and ARPL spectra as a reference, TCSPC data at a given PL wavelength could be collected with 2 nm resolution through diffraction of the cavity PL emission (Figures S12–14). TCSPC data were fit to a triple exponential with the instrument response function (IRF) deconvolution, and Figure 4b shows polariton PL lifetimes for the shortest component (τ_s_) resolved across the UPB/LPB for a sample with two different cavity detuning energy of −67 meV and −2 meV (Figure S11).

Interestingly, this short lifetime appears to reach a minimum of approximately 15 ps when the HH NPL exciton is resonant with the cavity and increases for UP and LP emission energies as the collection angle or in-plane momenta increases for both of the cavity detuning. Additionally, the average short component lifetime for both cavity detuning across all the LP (∼17 ps) and UP (∼21 ps) energies are 1.4–1.75X times shorter than for the bare thin film samples (30 ps) as shown in Table 1. The medium (τ_m_ ∼ 186–196 ps) and long (τ_l_ ∼ 1.5–1.6 ns) lifetime components under strong coupling remain largely unchanged across the various polariton PL emission energies and also from the uncoupled exciton lifetimes (Figure S16). Our results clearly indicate that the polariton PL lifetimes are not simply weighted by the Hopfield coefficients, which, for example, would result in a cavity photon lifetime of ∼160 fs near resonant angles corresponding to equal 50% excitonic and 50% photonic characters. Instead, the overall polariton PL lifetimes are significantly prolonged due to continuous population mixing with the dark state reservoir. Nonetheless, the range of measured polariton PL lifetimes for this rapid short component (τ_s_ = 15–26 ps) suggests that the transfer of population between the dark and polaritonic states has some dependence on the relative photonic versus excitonic character of the polariton at a given collection angle.

**Table 1:**
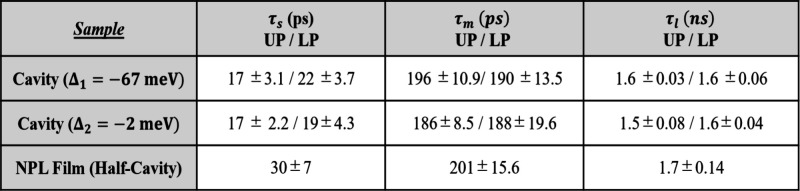
Summary of the Average Polariton Energy PL Lifetimes by Individual Components^a^

a Lifetime values by component (τ_s_ – short, τ_m_ – medium, τ_l_ – long) refer to averaged measurements across all UP/LP emission energies with errors given by the standard deviation for two cavity detuning. Three thin film (half-cavity) control measurements are reported as the average for comparison.

Identifying and comparing the individual PL lifetime components of the polariton and the uncoupled NPL exciton allows for further insight into how strong light–matter coupling affects NPL photophysics. The decrease of τ_s_ inside the cavity can be understood as a consequence of the moderately large number of coupled NPLs (∼16,000) which is large enough for the dark states to serve as a population reservoir for the polariton states, yet small enough to allow for an appreciable rate of transfer from these dark reservoir states to the polariton states. This appreciable reservoir to polariton transfer rate in NPL-cavity systems serves as an extra decay channel for the reservoir states, which adds onto the bare nonradiative decay rate to cause a decrease of the lifetime of the reservoir states. This decrease in reservoir lifetime is detected from the UP and LP due to a decrease in the population transfer rate from the reservoir to these polariton states as the reservoir population decays through time. This stands in contrast to many organic polariton systems which can have orders of magnitude more coupled molecules, which greatly reduces the rate of transfer from reservoir to polariton states,^27^ and consequentially do not have measurably different polariton PL lifetimes compared to the bare organic molecules.^31,74,75^ The trend of increasing UP PL lifetime as a function of energy in both Figure 4b also stands in contrast to previous works which found no polariton PL lifetime dependence on energy.^31,73^ While higher temporal and energy resolved pump–probe measurements are needed to track the population transfer rates between the individual states, one possible explanation of the trend is that the reduction of nonadiabatic coupling at higher angles and energies reduces the rate of population lost to other polariton states and thus increases the perceived PL lifetime of those higher energy states.

## Conclusions

By designing a high Q-factor FP cavity embedded with colloidal 2D CdSe NPLs, we were able to obtain great insight into how cavity parameters can be used to control excited polariton dynamics. Notably, while vibronic coupling allows for excited population to be upconverted from the LP through the dark states to the UP, high Q-factor cavities enable PL emission from the UP branch due to lower photon loss rates among both the UP states and the partially photonic quasi-dark states. Further, quantum dynamics simulations suggest that even higher Q cavities at room temperature will allow for a further enhancement of the UP population. The ability to control the upconversion of excited population from LP and dark states to the UP is a significant step toward enhancing forbidden photochemical reaction rates using strong light–matter coupling. For example, a photochemical reaction that has an uphill driving force outside the cavity, which is nominally forbidden (at equilibrium), could have its reaction rate significantly increased through coupling the molecular donor state to the cavity mode.^21,77^

In addition to controlling polariton excited populations, high Q-factor cavities allowed for measurements of PL lifetimes across the UP and LP states with energy resolved TCSPC. Comparing to average (amplitude) lifetimes of half-cavity NPL films (∼188 ps), we see an average polariton PL lifetime of ∼100 ps across two different cavity detuned samples primarily due to a decrease in the polariton short component lifetime, indicating a rapid exchange of population from the dark states to both the UP and LP via phonon coupling. The finding of a polariton lifetime for NPLs different from the free exciton lifetime is atypical for organic polariton systems but expected for these NPL polariton systems with fewer coupled emitters. This has important and direct implications for applications of polaritons in chemical systems. For example, the presence of a finite UP population lasting over 100 ps could eventually allow for polaritonic control over reactions that are initiated or catalyzed by a charge transfer event. Indeed, since many photochemical reactions occur on similar or shorter time scales^78,79^ polaritons could be used to control the product outcomes in a rational way, through driving reactions that are energetically forbidden in absence of the cavity. Altogether, the design of optical cavities that facilitate strong light–matter interactions with large Q-factors is a promising route to control distinct chemical transformations with nanocrystal-based polariton systems.

## Methods

### Synthesis of 4.5 Monolayer CdSe Nanoplatelets

The synthesis of 4.5 monolayer CdSe nanoplatelets was carried out using a previously reported method^80^ with slight modifications. 180 mg of anhydrous cadmium myristate, 30 mg of selenium powder, and 15 mL of 1-octadecene were added in a 100 mL 3-neck round-bottom flask. The contents of the flask were degassed at room temperature followed by degassing at 120 °C for 60 min. The mixture was then returned to a nitrogen environment, and the temperature was set to 240 °C. At 210 °C, the septum was removed from one neck, and 100 mg of cadmium acetate dihydrate were swiftly added to the solution. Once the temperature reached 240 °C, it was carefully maintained for 8 min. After which the reaction was quickly quenched with the aid of a heat gun followed by a water bath at 190 °C. A 2 mL volume of oleic acid was injected at 160 °C followed by injection of 15 mL of hexanes at room temperature. The nanoplatelets were precipitated by centrifugation at 3000 rpm for 10 min and were redispersed in 12 mL of hexanes. The solution was then allowed to sit overnight followed by centrifugation at 6000 rpm for 15 min. The pellet was discarded, and the nanoplatelets were kept in an air sealed glass vial.

### Microcavity Fabrication

The DBR mirror of the dielectric-metal microcavity was deposited via PE-CVD on Si substrates (1 cm × 1 cm) with 15.5 bilayers of alternating 60 nm Si_3_N_4_/85 nm SiO_2_ to form a 99.9% Bragg-reflector in the 450–550 nm region. For the bottom spacer layer, 200 nm of SiO_2_ was deposited via e-beam PVD or 200 nm of PMMA resist was spin coated onto the DBR mirror–both resulting in similar cavity performance. A concentrated and purified solution of 4.5 ML CdSe NPLs in hexane was drop-casted onto the spacer layer to approximately form a smooth 60 nm nanocrystal film. Another spacer layer of 200 nm SiO_2_ and the top 40 nm silver mirror were deposited via e-beam PVD to form the full 3λ/2n Fabry–Pérot microcavity.

### Angle Resolved Spectroscopy

For angle resolved spectra measurements, the cavity sample was mounted onto an MCL NanoH100 XY piezo stage (for raster scanning) that is integrated onto an epifluorescence microscope (Nikon TE-2000U). The sample was illuminated through the top silver mirror with an approximate focused 1 μm spot size using a 40*x*/0.6 NA objective (Nikon). Cavity emission was collected from the same objective with dichroic mirrors, and the back focal plane of the objective was relayed to the spectrometer with a tube lens for momentum or angle resolved spectra. For cold-temperature measurements, the sample was placed in a Montana Instruments CA50 cryostat with a 60*x*/0.7 NA objective. A broadband (450–750 nm) white light LED (Thorlabs) was used for all reflectance spectra, and several different laser pump sources were used for collecting angle resolved photoluminescence spectra across multiple experimental setups. A 5 MHz pulsed 485 nm laser diode (PicoQuant LDH-D 485) was used for steady-state room temperature spectra, while a CW 405 nm diode (PicoQuant LDH-D 405) was used for 100 K measurements. For upconversion experiments, a 2.5 MHz pulsed white-light continuum laser (FYLA Iceblink) with a band-pass filter was used for a broad 515–540 nm excitation of the LPB and a 2.44–2.56 eV band-pass filter (Chroma ET495/25x) was used to filter the PL from the UPB.

### Lifetime Measurements

A frequency-doubled Ti:sapphire laser (Coherent Mira-900) operating at 410 nm with 76 MHz repetition rate and <2 ps pulse durations was used for TCSPC lifetime measurements. An Acton SP-2500i dual exit port spectrometer (as shown in Figure 4a) was used to correlate the angle resolved spectra taken on the CCD with the wavelength-resolved lifetime collection taken with the single-photon APD detector (Micro Photon Devices MPD) connected to PicoHarp 300 (PicoQuant) time tagging (4 ps binning) electronics. The spectrometer grating (150 g/mm) along with the exit slits was used to obtain 2 nm wavelength resolution. To improve PL collection efficiency for TCSPC, the Fourier tube lens prior to the spectrometer was flipped out of the optical path and an imaging relay lens was placed into the optical path to provide a point image of the filtered PL (via grating) onto the single pixel APD detector. Lifetime components were obtained by deconvoluting the APD-limited 60 ps instrument response from the individual curves (Figures S11–S13) and fitted to a triple exponential function using the open-source MATLAB DecayFit (FluorTools) software. Lifetimes of three random positions along a half-cavity bare NPL film were also measured to compare against the polariton lifetimes.

### Quantum Dynamics Simulations

The GHTC Hamiltonian used for simulating the NPL-cavity system modeled *N* = 160 NPL molecules coupled to *K* = 40 cavity modes uniformly distributed among the in-plane wavevector component. A total of *K* = 80 cavity modes were used in upconversion simulations for enhanced resolution of the UPB dispersion. The steady-state of the populations from the L-MASH propagation were selected after 2 ps of continuous, incoherent driving of the ground state to the HH states with a per-molecule pump intensity of 6/*N* meV. The LP states in the upconversion simulations were pumped with a per-mode intensity of 6/*K* meV. The numerical time step was *dt* = 0.5 fs, and the PL spectra were averaged over 10,000 trajectories. The full details of the model and PL calculations can be found in the SI.

## Supporting Information Available

Additional details on cavity design, sample characterization, angle-resolved data fittings, quantum dynamical simulations, and lifetime measurements are provided. (PDF)

## Notes

A preprint version of this manuscript has been previously submitted to the ChemRxiv repository: Amin, M.; Koessler, E.R.; Morshed, O.; Awan, F.; Cogan, N.M.B.; Collison, R.; Tumiel, T.M.; Girten, W.; Leiter, C.S.; Vamivakas, A.N.; Huo, P.; Krauss, T.D. Cavity Controlled Upconversion in CdSe Nanoplatelet Polaritons. **2024**. *ChemRxiv*. DOI: 10.26434/chemrxiv-2023–4tshv-v2 (acccessed 2024/04/02).

## Abbreviations

CdSe: cadmium selenide
NPL: nanoplatelet
UP: upper polariton
LP: lower polariton
UPB/LPB: upper/lower polariton branch
PL: photoluminescence
Q-factor: quality factor
FP: Fabry-Pérot
HH: heavy hole
DBR: distributed Bragg reflector
ARR: angle resolved reflectance
ARPL: angle resolved photoluminescence
MASH: multistate mapping approach to surface hopping
L-MASH: Lindblad MASH
GHTC: generalized Holstein–Tavis–Cummings
TC: Tavis–Cummings
TCSPC: time correlated single photon counting

## References

[ref1] Efros A. L.; Brus L. E. Nanocrystal Quantum Dots: From Discovery to Modern Development. ACS Nano 2021, 15 (4), 6192–6210. 10.1021/acsnano.1c01399. 33830732

[ref2] Murray C. B.; Kagan C. R.; Bawendi M. G. Synthesis and Characterization of Monodisperse Nanocrystals and Close-Packed Nanocrystal Assemblies. Annu. Rev. Mater. Sci. 2000, 30 (1), 545–610. 10.1146/annurev.matsci.30.1.545.

[ref3] Schwartz T.; Hutchison J. A.; Genet C.; Ebbesen T. W. Reversible Switching of Ultrastrong Light-Molecule Coupling. Phys. Rev. Lett. 2011, 106 (19), 196405 10.1103/PhysRevLett.106.196405. 21668181

[ref4] Kowalewski M.; Mukamel S. Manipulating molecules with quantum light. Proc. Natl. Acad. Sci. U. S. A. 2017, 114 (13), 3278–3280. 10.1073/pnas.1702160114. 28302671 PMC5380056

[ref5] Munkhbat B.; Wersäll M.; Baranov D. G.; Antosiewicz T. J.; Shegai T. Suppression of photo-oxidation of organic chromophores by strong coupling to plasmonic nanoantennas. Science Advances 2018, 4 (7), eaas9552 10.1126/sciadv.aas9552. 29984306 PMC6035039

[ref6] Stranius K.; Hertzog M.; Börjesson K. Selective manipulation of electronically excited states through strong light–matter interactions. Nat. Commun. 2018, 9 (1), 2273 10.1038/s41467-018-04736-1. 29891958 PMC5995866

[ref7] Mandal A.; Huo P. Investigating New Reactivities Enabled by Polariton Photochemistry. J. Phys. Chem. Lett. 2019, 10 (18), 5519–5529. 10.1021/acs.jpclett.9b01599. 31475529

[ref8] Feist J.; Galego J.; Garcia-Vidal F. J. Polaritonic Chemistry with Organic Molecules. ACS Photonics 2018, 5 (1), 205–216. 10.1021/acsphotonics.7b00680.

[ref9] Ribeiro R. F.; Martínez-Martínez L. A.; Du M.; Campos-Gonzalez-Angulo J.; Yuen-Zhou J. Polariton chemistry: controlling molecular dynamics with optical cavities. Chemical Science 2018, 9 (30), 6325–6339. 10.1039/C8SC01043A. 30310561 PMC6115696

[ref10] Zeng H.; Pérez-Sánchez J. B.; Eckdahl C. T.; Liu P.; Chang W. J.; Weiss E. A.; Kalow J. A.; Yuen-Zhou J.; Stern N. P. Control of Photoswitching Kinetics with Strong Light–Matter Coupling in a Cavity. J. Am. Chem. Soc. 2023, 145 (36), 19655–19661. 10.1021/jacs.3c04254. 37643086

[ref11] Ahn W.; Triana J. F.; Recabal F.; Herrera F.; Simpkins B. S. Modification of ground-state chemical reactivity via light–matter coherence in infrared cavities. Science 2023, 380 (6650), 1165–1168. 10.1126/science.ade7147. 37319215

[ref12] Georgiou K.; Jayaprakash R.; Othonos A.; Lidzey D. G. Ultralong-Range Polariton-Assisted Energy Transfer in Organic Microcavities. Angew. Chem., Int. Ed. 2021, 60 (30), 16661–16667. 10.1002/anie.202105442. PMC8361947 33908681

[ref13] Coles D. M.; Somaschi N.; Michetti P.; Clark C.; Lagoudakis P. G.; Savvidis P. G.; Lidzey D. G. Polariton-mediated energy transfer between organic dyes in a strongly coupled optical microcavity. Nat. Mater. 2014, 13 (7), 712–719. 10.1038/nmat3950. 24793357

[ref14] Ribeiro R. F. Multimode polariton effects on molecular energy transport and spectral fluctuations. Communications Chemistry 2022, 5 (1), 48 10.1038/s42004-022-00660-0. 36697846 PMC9814737

[ref15] Xu D.; Mandal A.; Baxter J. M.; Cheng S.-W.; Lee I.; Su H.; Liu S.; Reichman D. R.; Delor M. Ultrafast imaging of polariton propagation and interactions. Nat. Commun. 2023, 14 (1), 3881 10.1038/s41467-023-39550-x. 37391396 PMC10313693

[ref16] Deng H.; Haug H.; Yamamoto Y. Exciton-polariton Bose–Einstein condensation. Rev. Mod. Phys. 2010, 82 (2), 1489–1537. 10.1103/RevModPhys.82.1489.

[ref17] Plumhof J. D.; Stöferle T.; Mai L.; Scherf U.; Mahrt R. F. Room-temperature Bose–Einstein condensation of cavity exciton–polaritons in a polymer. Nat. Mater. 2014, 13 (3), 247–252. 10.1038/nmat3825. 24317189

[ref18] Ghosh S.; Liew T. C. H. Quantum computing with exciton-polariton condensates. npj Quantum Information 2020, 6 (1), 16 10.1038/s41534-020-0244-x.

[ref19] Kim N. Y.; Yamamoto Y. Exciton-Polariton Quantum Simulators. In Quantum Simulations with Photons and Polaritons: Merging Quantum Optics with Condensed Matter Physics; Angelakis D. G., Ed.; Springer International Publishing: 2017; pp 91–121.

[ref20] Hutchison J. A.; Schwartz T.; Genet C.; Devaux E.; Ebbesen T. W. Modifying Chemical Landscapes by Coupling to Vacuum Fields. Angew. Chem., Int. Ed. 2012, 51 (7), 1592–1596. 10.1002/anie.201107033. 22234987

[ref21] Mandal A.; Krauss T. D.; Huo P. Polariton-Mediated Electron Transfer via Cavity Quantum Electrodynamics. J. Phys. Chem. B 2020, 124 (29), 6321–6340. 10.1021/acs.jpcb.0c03227. 32589846

[ref22] Mandal A.; Taylor M. A. D.; Weight B. M.; Koessler E. R.; Li X.; Huo P. Theoretical Advances in Polariton Chemistry and Molecular Cavity Quantum Electrodynamics. Chem. Rev. 2023, 123 (16), 9786–9879. 10.1021/acs.chemrev.2c00855. 37552606 PMC10450711

[ref23] Bhuyan R.; Mony J.; Kotov O.; Castellanos G. W.; Gómez Rivas J.; Shegai T. O.; Börjesson K. The Rise and Current Status of Polaritonic Photochemistry and Photophysics. Chem. Rev. 2023, 123 (18), 10877–10919. 10.1021/acs.chemrev.2c00895. 37683254 PMC10540218

[ref24] Weight B. M.; Krauss T. D.; Huo P. Investigating Molecular Exciton Polaritons Using Ab Initio Cavity Quantum Electrodynamics. J. Phys. Chem. Lett. 2023, 14 (25), 5901–5913. 10.1021/acs.jpclett.3c01294. 37343178 PMC10316409

[ref25] Thomas A.; Lethuillier-Karl L.; Nagarajan K.; Vergauwe R. M. A.; George J.; Chervy T.; Shalabney A.; Devaux E.; Genet C.; Moran J.; Ebbesen T. W. Tilting a ground-state reactivity landscape by vibrational strong coupling. Science 2019, 363 (6427), 615–619. 10.1126/science.aau7742. 30733414

[ref26] Garcia-Vidal F. J.; Ciuti C.; Ebbesen T. W. Manipulating matter by strong coupling to vacuum fields. Science 2021, 373 (6551), eabd0336 10.1126/science.abd0336. 34244383

[ref27] Pino J. d.; Feist J.; Garcia-Vidal F. J. Quantum theory of collective strong coupling of molecular vibrations with a microcavity mode. New J. Phys. 2015, 17 (5), 053040 10.1088/1367-2630/17/5/053040.

[ref28] Litinskaya M.; Reineker P.; Agranovich V. M. Fast polariton relaxation in strongly coupled organic microcavities. J. Lumin. 2004, 110 (4), 364–372. 10.1016/j.jlumin.2004.08.033.

[ref29] Fassioli F.; Park K. H.; Bard S. E.; Scholes G. D. Femtosecond Photophysics of Molecular Polaritons. J. Phys. Chem. Lett. 2021, 12 (46), 11444–11459. 10.1021/acs.jpclett.1c03183. 34792371

[ref30] Scholes G. D.; DelPo C. A.; Kudisch B. Entropy Reorders Polariton States. J. Phys. Chem. Lett. 2020, 11 (15), 6389–6395. 10.1021/acs.jpclett.0c02000. 32678609

[ref31] Xiang B.; Ribeiro R. F.; Chen L.; Wang J.; Du M.; Yuen-Zhou J.; Xiong W. State-Selective Polariton to Dark State Relaxation Dynamics. J. Phys. Chem. A 2019, 123 (28), 5918–5927. 10.1021/acs.jpca.9b04601. 31268708

[ref32] Khazanov T.; Gunasekaran S.; George A.; Lomlu R.; Mukherjee S.; Musser A. J. Embrace the darkness: An experimental perspective on organic exciton–polaritons. Chemical Physics Reviews 2023, 4 (4), 041305 10.1063/5.0168948.

[ref33] Michail E.; Rashidi K.; Liu B.; He G.; Menon V. M.; Sfeir M. Y. Addressing the Dark State Problem in Strongly Coupled Organic Exciton-Polariton Systems. Nano Lett. 2024, 24 (2), 557–565. 10.1021/acs.nanolett.3c02984. 38179964

[ref34] Groenhof G.; Climent C.; Feist J.; Morozov D.; Toppari J. J. Tracking Polariton Relaxation with Multiscale Molecular Dynamics Simulations. J. Phys. Chem. Lett. 2019, 10 (18), 5476–5483. 10.1021/acs.jpclett.9b02192. 31453696 PMC6914212

[ref35] Qiu L.; Mandal A.; Morshed O.; Meidenbauer M. T.; Girten W.; Huo P.; Vamivakas A. N.; Krauss T. D. Molecular Polaritons Generated from Strong Coupling between CdSe Nanoplatelets and a Dielectric Optical Cavity. J. Phys. Chem. Lett. 2021, 12 (20), 5030–5038. 10.1021/acs.jpclett.1c01104. 34018749

[ref36] Du M.; Campos-Gonzalez-Angulo J. A.; Yuen-Zhou J. Nonequilibrium effects of cavity leakage and vibrational dissipation in thermally activated polariton chemistry. J. Chem. Phys. 2021, 154 (8), 084108 10.1063/5.0037905. 33639750

[ref37] Koner A.; Du M.; Pannir-Sivajothi S.; Goldsmith R. H.; Yuen-Zhou J. A path towards single molecule vibrational strong coupling in a Fabry–Pérot microcavity. Chemical Science 2023, 14 (28), 7753–7761. 10.1039/D3SC01411H. 37476723 PMC10355109

[ref38] Flatten L. C.; Christodoulou S.; Patel R. K.; Buccheri A.; Coles D. M.; Reid B. P. L.; Taylor R. A.; Moreels I.; Smith J. M. Strong Exciton–Photon Coupling with Colloidal Nanoplatelets in an Open Microcavity. Nano Lett. 2016, 16 (11), 7137–7141. 10.1021/acs.nanolett.6b03433. 27737546

[ref39] Winkler J. M.; Rabouw F. T.; Rossinelli A. A.; Jayanti S. V.; McPeak K. M.; Kim D. K.; le Feber B.; Prins F.; Norris D. J. Room-Temperature Strong Coupling of CdSe Nanoplatelets and Plasmonic Hole Arrays. Nano Lett. 2019, 19 (1), 108–115. 10.1021/acs.nanolett.8b03422. 30516054 PMC6578340

[ref40] Shlesinger I.; Monin H.; Moreau J.; Hugonin J.-P.; Dufour M.; Ithurria S.; Vest B.; Greffet J.-J. Strong Coupling of Nanoplatelets and Surface Plasmons on a Gold Surface. ACS Photonics 2019, 6 (11), 2643–2648. 10.1021/acsphotonics.9b01133.

[ref41] Yang H.; Zhang L.; Xiang W.; Lu C.; Cui Y.; Zhang J. Ultralow Threshold Room Temperature Polariton Condensation in Colloidal CdSe/CdS Core/Shell Nanoplatelets. Advanced Science 2022, 9 (18), 2200395 10.1002/advs.202200395. 35466544 PMC9218774

[ref42] Freire-Fernández F.; Sinai N. G.; Hui Tan M. J.; Park S.-M.; Koessler E. R.; Krauss T.; Huo P.; Odom T. W. Room-Temperature Polariton Lasing from CdSe Core-Only Nanoplatelets. ACS Nano 2024, 18 (23), 15177–15184. 10.1021/acsnano.4c03164. 38808728

[ref43] Ithurria S.; Tessier M. D.; Mahler B.; Lobo R. P. S. M.; Dubertret B.; Efros A. L. Colloidal nanoplatelets with two-dimensional electronic structure. Nat. Mater. 2011, 10 (12), 936–941. 10.1038/nmat3145. 22019946

[ref44] Tessier M. D.; Javaux C.; Maksimovic I.; Loriette V.; Dubertret B. Spectroscopy of Single CdSe Nanoplatelets. ACS Nano 2012, 6 (8), 6751–6758. 10.1021/nn3014855. 22783952

[ref45] Geiregat P.; Rodá C.; Tanghe I.; Singh S.; Di Giacomo A.; Lebrun D.; Grimaldi G.; Maes J.; Van Thourhout D.; Moreels I.; et al. Localization-limited exciton oscillator strength in colloidal CdSe nanoplatelets revealed by the optically induced stark effect. Light: Science & Applications 2021, 10 (1), 112 10.1038/s41377-021-00548-z. PMC8165098 34054127

[ref46] Ithurria S.; Dubertret B. Quasi 2D Colloidal CdSe Platelets with Thicknesses Controlled at the Atomic Level. J. Am. Chem. Soc. 2008, 130 (49), 16504–16505. 10.1021/ja807724e. 19554725

[ref47] Pandya R.; Ashoka A.; Georgiou K.; Sung J.; Jayaprakash R.; Renken S.; Gai L.; Shen Z.; Rao A.; Musser A. J. Tuning the Coherent Propagation of Organic Exciton-Polaritons through Dark State Delocalization. Advanced Science 2022, 9 (18), 2105569 10.1002/advs.202105569. 35474309 PMC9218652

[ref48] Koessler E. R.; Mandal A.; Huo P. Incorporating Lindblad decay dynamics into mixed quantum-classical simulations. J. Chem. Phys. 2022, 157 (6), 064101 10.1063/5.0099922. 35963729

[ref49] Morshed O.; Amin M.; Cogan N. M. B.; Koessler E. R.; Collison R.; Tumiel T. M.; Girten W.; Awan F.; Mathis L.; Huo P.; et al. Room-temperature strong coupling between CdSe nanoplatelets and a metal–DBR Fabry–Pérot cavity. J. Chem. Phys. 2024, 161 (1), 014710 10.1063/5.0210700. 38953450

[ref50] Neuman T.; Aizpurua J. Origin of the asymmetric light emission from molecular exciton–polaritons. Optica 2018, 5 (10), 1247–1255. 10.1364/OPTICA.5.001247.

[ref51] Müller K.; Fischer K. A.; Rundquist A.; Dory C.; Lagoudakis K. G.; Sarmiento T.; Kelaita Y. A.; Borish V.; Vučković J. Ultrafast Polariton-Phonon Dynamics of Strongly Coupled Quantum Dot-Nanocavity Systems. Physical Review X 2015, 5 (3), 031006 10.1103/PhysRevX.5.031006.

[ref52] Coles D. M.; Michetti P.; Clark C.; Adawi A. M.; Lidzey D. G. Temperature dependence of the upper-branch polariton population in an organic semiconductor microcavity. Phys. Rev. B 2011, 84 (20), 205214 10.1103/PhysRevB.84.205214.

[ref53] Lidzey D. G.; Fox A. M.; Rahn M. D.; Skolnick M. S.; Agranovich V. M.; Walker S. Experimental study of light emission from strongly coupled organic semiconductor microcavities following nonresonant laser excitation. Phys. Rev. B 2002, 65 (19), 195312 10.1103/PhysRevB.65.195312.

[ref54] Michetti P.; La Rocca G. C. Simulation of J-aggregate microcavity photoluminescence. Phys. Rev. B 2008, 77 (19), 195301 10.1103/PhysRevB.77.195301.

[ref55] Wenus J.; Connolly L. G.; Lidzey D. G. New organic materials and microcavity structures for strong exciton-photon coupling. physica status solidi (c) 2005, 2 (11), 3899–3902. 10.1002/pssc.200562041.

[ref56] Houdré R.; Weisbuch C.; Stanley R. P.; Oesterle U.; Pellandini P.; Ilegems M. Measurement of Cavity-Polariton Dispersion Curve from Angle-Resolved Photoluminescence Experiments. Phys. Rev. Lett. 1994, 73 (15), 2043–2046. 10.1103/PhysRevLett.73.2043. 10056957

[ref57] Tinkler L.; Walker P. M.; Clarke E.; Krizhanovskii D. N.; Bastiman F.; Durska M.; Skolnick M. S. Design and characterization of high optical quality InGaAs/GaAs/AlGaAs-based polariton microcavities. Appl. Phys. Lett. 2015, 106 (2), 021109 10.1063/1.4905907.

[ref58] Skolnick M. S.; Fisher T. A.; Whittaker D. M. Strong coupling phenomena in quantum microcavity structures. Semicond. Sci. Technol. 1998, 13 (7), 645 10.1088/0268-1242/13/7/003.

[ref59] Mannouch J. R.; Richardson J. O. A mapping approach to surface hopping. J. Chem. Phys. 2023, 158 (10), 104111 10.1063/5.0139734. 36922129

[ref60] Runeson J. E.; Manolopoulos D. E. A multi-state mapping approach to surface hopping. J. Chem. Phys. 2023, 159 (9), 094115 10.1063/5.0158147. 37675848

[ref61] Tichauer R. H.; Feist J.; Groenhof G. Multi-scale dynamics simulations of molecular polaritons: The effect of multiple cavity modes on polariton relaxation. J. Chem. Phys. 2021, 154 (10), 104112 10.1063/5.0037868. 33722041

[ref62] Hobson P. A.; Barnes W. L.; Lidzey D. G.; Gehring G. A.; Whittaker D. M.; Skolnick M. S.; Walker S. Strong exciton–photon coupling in a low-Q all-metal mirror microcavity. Appl. Phys. Lett. 2002, 81 (19), 3519–3521. 10.1063/1.1517714.

[ref63] Lidzey D. G.; Bradley D. D. C.; Virgili T.; Armitage A.; Skolnick M. S.; Walker S. Room Temperature Polariton Emission from Strongly Coupled Organic Semiconductor Microcavities. Phys. Rev. Lett. 1999, 82 (16), 3316–3319. 10.1103/PhysRevLett.82.3316.

[ref64] Coles D. M.; Grant R. T.; Lidzey D. G.; Clark C.; Lagoudakis P. G. Imaging the polariton relaxation bottleneck in strongly coupled organic semiconductor microcavities. Phys. Rev. B 2013, 88 (12), 121303 10.1103/PhysRevB.88.121303.

[ref65] Graf A.; Tropf L.; Zakharko Y.; Zaumseil J.; Gather M. C. Near-infrared exciton-polaritons in strongly coupled single-walled carbon nanotube microcavities. Nat. Commun. 2016, 7 (1), 13078 10.1038/ncomms13078. 27721454 PMC5062498

[ref66] Lüttgens J. M.; Berger F. J.; Zaumseil J. Population of Exciton–Polaritons via Luminescent sp3 Defects in Single-Walled Carbon Nanotubes. ACS Photonics 2021, 8 (1), 182–193. 10.1021/acsphotonics.0c01129. 33506074 PMC7821305

[ref67] Herrera F.; Spano F. C. Absorption and photoluminescence in organic cavity QED. Phys. Rev. A 2017, 95 (5), 053867 10.1103/PhysRevA.95.053867.

[ref68] Varshni Y. P. Temperature dependence of the energy gap in semiconductors. Physica 1967, 34 (1), 149–154. 10.1016/0031-8914(67)90062-6.

[ref69] Antolinez F. V.; Rabouw F. T.; Rossinelli A. A.; Keitel R. C.; Cocina A.; Becker M. A.; Norris D. J. Trion Emission Dominates the Low-Temperature Photoluminescence of CdSe Nanoplatelets. Nano Lett. 2020, 20 (8), 5814–5820. 10.1021/acs.nanolett.0c01707. 32589429

[ref70] Ayari S.; Quick M. T.; Owschimikow N.; Christodoulou S.; Bertrand G. H. V.; Artemyev M.; Moreels I.; Woggon U.; Jaziri S.; Achtstein A. W. Tuning trion binding energy and oscillator strength in a laterally finite 2D system: CdSe nanoplatelets as a model system for trion properties. Nanoscale 2020, 12 (27), 14448–14458. 10.1039/D0NR03170D. 32618327

[ref71] Rana F.; Koksal O.; Jung M.; Shvets G.; Vamivakas A. N.; Manolatou C. Exciton-Trion Polaritons in Doped Two-Dimensional Semiconductors. Phys. Rev. Lett. 2021, 126 (12), 127402 10.1103/PhysRevLett.126.127402. 33834815

[ref72] Dufferwiel S.; Lyons T. P.; Solnyshkov D. D.; Trichet A. A. P.; Withers F.; Schwarz S.; Malpuech G.; Smith J. M.; Novoselov K. S.; Skolnick M. S.; et al. Valley-addressable polaritons in atomically thin semiconductors. Nat. Photonics 2017, 11 (8), 497–501. 10.1038/nphoton.2017.125.

[ref73] Mony J.; Hertzog M.; Kushwaha K.; Börjesson K. Angle-Independent Polariton Emission Lifetime Shown by Perylene Hybridized to the Vacuum Field Inside a Fabry–Pérot Cavity. J. Phys. Chem. C 2018, 122 (43), 24917–24923. 10.1021/acs.jpcc.8b07283. PMC6234983 30450150

[ref74] Schwartz T.; Hutchison J. A.; Léonard J.; Genet C.; Haacke S.; Ebbesen T. W. Polariton Dynamics under Strong Light–Molecule Coupling. ChemPhysChem 2013, 14 (1), 125–131. 10.1002/cphc.201200734. 23233286

[ref75] Canaguier-Durand A.; Genet C.; Lambrecht A.; Ebbesen T. W.; Reynaud S. Non-Markovian polariton dynamics in organic strong coupling. European Physical Journal D 2015, 69 (1), 24 10.1140/epjd/e2014-50539-x.

[ref76] Laitz M.; Kaplan A. E. K.; Deschamps J.; Barotov U.; Proppe A. H.; García-Benito I.; Osherov A.; Grancini G.; deQuilettes D. W.; Nelson K. A.; et al. Uncovering temperature-dependent exciton-polariton relaxation mechanisms in hybrid organic-inorganic perovskites. Nat. Commun. 2023, 14 (1), 2426 10.1038/s41467-023-37772-7. 37105984 PMC10140020

[ref77] Mauro L.; Caicedo K.; Jonusauskas G.; Avriller R. Charge-transfer chemical reactions in nanofluidic Fabry-P\’erot cavities. Phys. Rev. B 2021, 103 (16), 165412 10.1103/PhysRevB.103.165412.

[ref78] Xu J.-Y.; Tong X.; Yu P.; Wenya G. E.; McGrath T.; Fong M. J.; Wu J.; Wang Z. M. Ultrafast Dynamics of Charge Transfer and Photochemical Reactions in Solar Energy Conversion. Advanced Science 2018, 5 (12), 1800221 10.1002/advs.201800221. 30581691 PMC6299728

[ref79] Gopidas K. R.; Bohorquez M.; Kamat P. V. Photophysical and photochemical aspects of coupled semiconductors: charge-transfer processes in colloidal cadmium sulfide-titania and cadmium sulfide-silver(I) iodide systems. J. Phys. Chem. 1990, 94 (16), 6435–6440. 10.1021/j100379a051.

[ref80] Bertrand G. H. V.; Polovitsyn A.; Christodoulou S.; Khan A. H.; Moreels I. Shape control of zincblende CdSe nanoplatelets. Chem. Commun. 2016, 52 (80), 11975–11978. 10.1039/C6CC05705E. 27722289

