# Supplementary material for: Cavity Controlled Upconversion in CdSe Nanoplatelet Polaritons
Source: ACS Nano. 2024 Jul 30;18(32):21388–98. doi: 10.1021/acsnano.4c05871 (PMC11328175; doi:10.1021/acsnano.4c05871)
Supplement: Supplementary file 1 — nn4c05871_si_001.pdf [file nn4c05871_si_001.pdf]

Supporting Information for

Cavity Controlled Upconversion in CdSe Nanoplatelet Polaritons

Mitesh Amin[a§], Eric R. Koessler[b§], Ovishek Morshed[a], Farwa Awan[b], Nicole M.B. Cogan[b], Robert Collison[a], Trevor M. Tumiel[b], William Girten[b], Christopher Leiter[c], A. Nickolas Vamivakas[a,d], Pengfei Huo[b,a], Todd D. Krauss[b,a]**

[a]The Institute of Optics, University of Rochester, Rochester, NY 14627, USA

[b]Department of Chemistry, University of Rochester, Rochester, NY 14627, USA

[c]Department of Chemistry, Regis University, Denver, CO 80221, USA

[d]Department of Physics and Astronomy, University of Rochester, Rochester, NY 14627, USA

[§]M.A. and E.R.K. contributed equally to this work.

*Corresponding authors: Pengfei Huo, Todd D. Krauss
Email: pengfei.huo@rochester.edu, todd.krauss@rochester.edu

Table of Contents

Cavity Design & Fabrication **3**

Number of Coupled NPLs **4**

Fitting Angle-Resolved Spectra **4**

Quantum Dynamical Simulations **5**

Supplemental Figures **10**
Fig. S1. Microcavity Design 10
Fig. S2. Sample Characterization 11
Fig. S3. Coupled Harmonic Oscillator Model Fitting 12
Fig. S4. Strong Coupling for Blue-Detuned Cavity 13
Fig. S5. 295 K vs. 100 K Strong Coupling 14
Fig. S6. Comparison of LMASH vs HEOM Models 15
Fig. S7. Polariton State Trajectory 16
Fig. S8. Experiment vs. Theory (100K) 17
Fig. S9. Fitted Data for Upconversion 18
Fig. S10. Resonant LPB Excitation 19
Fig. S11. ARR & ARPL for Lifetime Measurements 20
Fig. S12. Thin Film Lifetime Curves 21
Fig. S13. Polariton Lifetime Curves (Δ = -67 meV) 22
Fig. S14. Polariton Lifetime Curves (Δ = -2 meV) 23
Fig. S15. Triple Exponential IRF Deconvolution Fits (Short Times) 24
Fig. S16. Fitted Lifetime Components 25
Table S1. Thin Film Components 26
Table S2. Polariton Lifetimes Components (Δ = -67 meV) 27
Table S3. Polariton Lifetimes Components (Δ = -2 meV) 28
Table S4. Parameters of the GHTC model 29

References **30**

Supporting Information Text

Cavity Design. Various Fabry-Pérot cavity designs with CdSe NPLs as the active layer were explored and optimized for high Q-factors resonant with the 2.42 eV heavy-hole transition using transfer matrix method (TMM) [1] to simulate the overall thin film reflectance of the microcavity. Fig. S1 shows the calculated reflectance curves and the corresponding layer thicknesses for three cavity configurations: 1) Ag-NPL-Ag 2) Ag-NPL-DBR 3) Ag-SiO_2-NPL-SiO_2-DBR. The high index (n ~ 1.8 - 2.1) CdSe NPL film sandwiched between two silver mirrors yields a lossy cavity with Q-factors ranging from 15 – 30, however replacing the bottom metal mirror with a 99.9% highly-reflective 15.5 layers of SiO_2/Si_3N_4 (85 nm/61 nm) DBR mirror improves the Q-factor to about 60 – 80. Furthermore, by adding two symmetric SiO_2 (n ~ 1.46) spacer layers around the CdSe NPLs, the effective index of the cavity is reduced, and this thicker 3λ/2 geometry yields a significantly higher Q-factor in the range of 300 – 320. In addition to TMM calculations, FDTD simulations for the various cavity designs using Ansys Lumerical software were performed to model the localized electric field intensity at the NPL active layer inside the cavity.

Cavity Sample Preparation & Characterization. White-light reflectivity measurements and film thickness characterization were performed using Filmetrics F20 system with a 10X microscope objective (Fig. S2). Prior to depositing NPL films onto the DBR mirrors, film uniformity and thickness were optimized for both the metal-DBR cavities (without and with SiO_2 spacers) by drop-casting varying concentrations of 2x ethanol washed NPLs dispersed in hexane onto test silicon chips and obtaining thickness values from the Filmetrics system (n ~ 1.8 - 2.1). For the higher Q cavity, the target concentrations were then deposited onto the DBR + SiO_2 spacer layer stack and the LH/HH absorption transitions of the 4.5 ML NPLs were confirmed in reflectivity measurements as shown in Fig. S2C. Rather than using an entirely filled SiO_2/PMMA $3\lambda/2$ thick cavity to characterize the Q-factor of our system, we measured the Q-factor of samples containing active layer of NPLs to account for any additional scattering losses. This was done by taking a linecut at normal incidence ($\theta = 0°$) from the angle-resolved reflectance (ARR) spectra and fitting the resonance to a Lorentzian fit for a sample position that is highly red-

detuned at 2.31 eV from the 2.42 eV exciton transition as shown in Fig. S2D to obtain a Q ~ 300. Similarly, for the cavity without any spacers, a lower Q of approximately 60 was obtained for a highly red-detuned sample. The ARR spectra for this position defined in the angle range given by the 0.6 NA objective did not show any strong coupling.

Estimation of NPL Nanocrystals Strongly Coupled Inside Cavity. The Rabi splitting energy $\hbar\Omega_R$ is given by:

$$\hbar\Omega_R = \sqrt{N}\sqrt{\frac{2\hbar\omega_{\mathbf{c}}}{\epsilon V}} \cdot \mu$$

where N is the number of coupled NPLs, $\hbar\omega_{\mathbf{c}}$ is the cavity resonance energy of 2.4 eV, ϵ is the effective permittivity inside the cavity estimated to be $2.72\epsilon_o$, and transition dipole strength, $\mu = 20D$, for the 4.5 ML CdSe NPLs [2, 3]. We approximate the laser excitation mode volume, V, as the product of the area given by beam waist radius, w_o, and the Rayleigh distance as the thickness, $t_{NPL} = 60\ nm$, of the active NPL layer.

$$V = D^2 t_{NPL} = (2w_o)^2 t_{NPL} = (2\lambda_{485nm})^2 t_{NPL} = 0.056\ um^3$$

For a Rabi splitting energy of 40 meV, we calculate approximately 16,320 NPLs coupled inside the mode volume, which is in reasonable agreement of 32,000 NPLs estimated from geometric calculations given by 100% NPL packing density in the 60 nm thick film where each NPL has a surface area of 22 nm x 15 nm from TEM characterization and a NPL-to-NPL interspace stacking distance of 5.4 nm resulting from long oleic acid ligand chains [4]. The drop casting film preparation method likely results in additional voids from imperfect stacking, likely leading to lower NPL packing densities (~50%) which can account for the discrepancies between the two approximations.

Fitting Angle-Resolved Spectra. The lower (E_-) and upper (E_+) polariton branches were extracted by taking the minimum reflectance values along several vertical angle of incidence (θ) linecuts in the ARR spectra as shown in Fig. S3 and fitted to the coupled Harmonic oscillator model [5] to determine the Rabi splitting energy ($\hbar\Omega$), cavity energy at

0° (E_C), and the cavity-detuning energy (Δ), with exciton HH transition energy $E_X = 2.4218\ eV\ (295\ K), 2.46\ eV\ (100\ K)$:

$$E_{\pm}(\theta) = \frac{1}{2}\left[E_X + E_C(1 + \frac{1}{2}\tan^2\theta)\right] \pm \frac{1}{2}\sqrt{\left[E_C(1 + \frac{1}{2}\tan^2\theta) - E_X\right]^2 + (\hbar\Omega)^2}$$

$$\Delta(\theta) = E_C(1 + \frac{1}{2}tan^2\theta) - E_X$$

The fitted values and curves are overlaid onto both the ARR and ARPL plots to show PL emission from both the UP and LP observed under varying detuning energy across multiple samples.

Quantum Dynamical Simulations. The NPL-cavity system was modeled using the generalized Holstein-Tavis-Cummings (GHTC) model which has been previously used to study light-matter hybrid systems in Fabry-Perot cavities [6-9]. The GHTC Hamiltonian models several matter excitations coupled to several angle-dependent photonic modes which allows for the calculation of angle-resolved properties of polariton systems that contain a reservoir of dark states. The GHTC Hamiltonian can be expressed as

$$H_{GHTC} = H_{NPL} + H_{ph} + H_I$$

where H_{NPL} describes the exciton states of N independent nanoplatelets, H_{ph} is the Hamiltonian for the quantized cavity modes, and H_I describes the matter-cavity interactions (between H_{NPL} and H_{ph}). The NPL Hamiltonian is modeled as

$$H_{NPL} = \sum_{j=1}^{N} \hbar\omega_{HH}\sigma_{HH,j}^{\dagger}\,\sigma_{HH,j} + H_{ep}$$

where ω_{HH} is the frequency of the heavy hole (HH) exciton state, $\sigma_{HH,j}$ is the lowering operators of the HH of the jth NPL, and N is the number of coupled NPLs. Further, H_{ep} describes the Holstein exciton-phonon couplings as

$$H_{ep}(R) = \sum_{j=1}^{N}\sum_{v}\frac{P_{v,j}^2}{2} + \frac{1}{2}\omega_{v,j}^2\left(R_{v,j} - \sqrt{\frac{2\hbar S_{v,j}}{\omega_{v,j}}}\sigma_{HH,j}^{\dagger}\sigma_{HH,j}\right)^2$$

where $R_{v,j}$ and $P_{v,j}$ are the position and momenta of the vth phonon mode of the jth NPL, respectively, $\omega_{v,j}$ is the frequency of the phonon mode, and $S_{v,j}$ is the exciton-phonon coupling strength of the phonon mode. The total reorganization energy of each NPL HH is $\lambda_{\mathrm{HH}} = \sum_{v}\hbar\omega_{v,j}S_{v,j}$. We model the exciton-phonon coupling using a discretized super-ohmic spectral density (5) fitted from recent *ab initio* simulations [10] with 200 discrete bath modes and a total reorganization energy of $\lambda_{\mathrm{HH}} = 5$ meV. The photonic Hamiltonian is expressed as

$$H_{ph} = \sum_{\mathbf{k}}\hbar\omega_{\mathbf{k}}\left(a_{\mathbf{k}}^{\dagger}a_{\mathbf{k}} + \frac{1}{2}\right)$$

where $\omega_{\mathbf{k}}$ is the frequency of the kth angular mode of the Fabry-Perot cavity and $a_{\mathbf{k}}$ is the annihilation operator of the kth mode. For Fabry-Perot cavities, the cavity frequency $\omega_{\mathbf{k}}$ can be expressed as

$$\hbar\omega_{\mathbf{k}} = \hbar\omega_{\mathbf{c}}\sqrt{1 + \left(\frac{k_{\parallel}}{k_{\perp}}\right)^2} = \hbar\omega_{\mathbf{c}}\sqrt{1 + (\tan\theta)^2}$$

where $\hbar\omega_{\mathbf{c}} = \frac{\hbar c k_{\perp}}{n_c}$ is the cavity energy at normal incidence, c is the speed of light, n_c is the refractive index inside the cavity, $k_{\parallel}$ and $k_{\perp}$ are the wavevector components of the photon mode which are parallel and perpendicular to the cavity mirrors, respectively, and θ is the angle of incidence. For the relevant optical frequencies near the HH energy, the

perpendicular wavevector component $k_\perp$ is fixed and only the parallel component $k_\parallel$ will vary as a function of incident angle.

The light-matter coupling term is expressed as

$$H_I = \sum_{j=1}^{N} \sum_{\mathbf{k}} \hbar g_c \left(\sigma_{HH,j}^\dagger a_{\mathbf{k}} e^{i\mathbf{x}_j \cdot \mathbf{k}} + \sigma_{HH,j} a_{\mathbf{k}}^\dagger e^{-i\mathbf{x}_j \cdot \mathbf{k}} \right)$$

where $\mathbf{x}_j$ is the center-of-mass position of the jth NPL, $\mathbf{k}$ is the wavevector of a cavity mode, and g_c is the single molecule light-matter coupling strength. The collective Rabi splitting in this model is $\Omega_R = 2\sqrt{N} g_c$. In this work, we use $N = 160$ molecules and $K = 40$ cavity modes to keep the computational expense tractable. A summary of the simulation parameters is provided in Table S4. Note that we have assumed each individual coupling strength between the cavity modes and each NPL to be identical.

The population dynamics of the GHTC model with cavity loss were propagated using the multi-state mapping approach to surface hopping (MASH) approach [11, 12] that has been modified to include Lindblad dynamics (L-MASH). The Lindblad modifications to MASH are similar to those of the Lindblad mean-field Ehrenfest (L-MFE) method [13] and are briefly described here. The dissipative dynamics of Lindblad jump operators of the form $|0\rangle\langle 1|$ modify the propagation of the wavefunction coefficients which can be expressed as

$$c_1(t + dt) = e^{i\theta_{r1}} e^{i\varphi_1} \sqrt{e^{-\Gamma \mathrm{dt}} |c_1(t)|^2 + (1 - e^{-\Gamma \mathrm{dt}}) Z}$$

$$c_0(t + dt) = e^{i\theta_{r0}} e^{i\varphi_0} \sqrt{|c_0(t)|^2 + (1 - e^{-\Gamma \mathrm{dt}})(|c_1(t)|^2 - Z)}$$

where c_0 and c_1 are the wavefunction coefficients of states 0 and 1, respectively, φ_0 and φ_1 are the complex phases of the coefficients at time t, Γ is the loss rate, $Z = \left(N_s - \sum_{n=1}^{N_s} \frac{1}{n}\right) / \left({N_s}^2 - N_s\right)$ is the effective zero-point energy of the system's population

with number of states N_s, and θ_{r0} and θ_{r1} are random phases sampled from the uniform distribution

$$\mathrm{P}(\theta_{ri}) = \frac{1}{2\Delta\theta_{ri}}, \quad -\Delta\theta_{ri} \leq \theta_{ri} < \Delta\theta_{ri}, \quad i = 0,1$$

where the bounds of the uniform distributions $\Delta\theta_{r0}$ and $\Delta\theta_{r1}$ are determined as

$$\frac{\sin \Delta\theta_{r1}}{\Delta\theta_{r1}} = \frac{e^{-\Gamma \mathrm{dt}/2}|c_1(t)|}{\sqrt{e^{-\Gamma \mathrm{dt}}|c_1(t)|^2 + (1 - e^{-\Gamma \mathrm{dt}})Z}}$$

$$\frac{\sin \Delta\theta_{r0}}{\Delta\theta_{r0}} = \frac{|c_0(t)|}{\sqrt{|c_0(t)|^2 + (1 - e^{-\Gamma \mathrm{dt}})(|c_1(t)|^2 - Z)}}$$

which can be numerically solved to find the bounds.

A comparison between L-MASH and HEOM [14] is shown in Fig. S6 for a GHTC model with 6 molecules and 3 cavity modes. The MASH method populations match very close to those of HEOM without loss in Fig. S6A and are nearly identical at later times, which is expected based on the ability of MASH to guarantee the correct steady-state thermal Boltzmann populations for ergodic systems [11]. The results with cavity loss in Fig. S6B similarly show very close agreement between the L-MASH an HEOM populations.

The non-adiabatic coupling presented in Fig. 2C in the main text is based on a numerical calculation of the coupling based on the polariton eigenstate expansion coefficients in the diabatic-Fock basis at different timesteps [15, 16]. The approximate non-adiabatic coupling V_{nm} between polariton states n and m over a small timestep Δt is

$$V_{nm} = \frac{-i\hbar}{2\Delta t} \sum_a \left(C^*{}_{na}(t) C_{ma}(t + \Delta t) - C^*{}_{na}(t + \Delta t) C_{ma}(t) \right)$$

where $C_{na} = \langle a|\psi_n\rangle$ is polariton expansion coefficient for polariton state n and diabatic-Fock state a. For Fig. 2C, the contribution of coupling to polariton state n from the cavity mode index $\tilde{k}$ near 8° was summed over all other polariton states m to yield the total non-adiabatic coupling contribution $\tilde{V}_n$ near 8° which is expressed as

$$\tilde{V}_n = \frac{\hbar}{2\Delta t}\sum_m |C^*{}_{n\tilde{k}}(t)C_{m\tilde{k}}(t+\Delta t) - C^*{}_{n\tilde{k}}(t+\Delta t)C_{m\tilde{k}}(t)|$$

The photoluminescence intensity at a steady-state time t_{ss} for a given energy and angle was calculated as

$$\mathrm{PL}(E,k) = \sum_n \frac{\rho_n(t_{ss})|\langle k|\psi_n\rangle|^2 \Gamma \sum_{k'}|\langle k'|\psi_n\rangle|^2}{2((E-E_n)^2 + (\Gamma\sum_{k'}|\langle k'|\psi_n\rangle|^2)^2/4)}$$

where k is the cavity mode index of the angle, Γ is the cavity loss rate, $\rho_n(t_{ss})$ is the steady-state population of polariton state n, $\{k'\}$ are the set of all cavity mode indices, and E_n is the eigenenergy of polariton state n. For the PL spectra in this work, the active states in the adiabatic basis were used as the population estimators for faster convergence with fewer trajectories. The total photonic character of the polariton states in Fig. S7 is $\sum_{k'}|\langle k'|\psi_n\rangle|^2$ for polariton state n.

For the upconversion simulations in Fig. 3, polariton eigenstates with energies at 2.4 eV and below were incoherently pumped with a rate weighted by their photonic character in order to simulate the direct laser pumping to the LP in the upconversion experiments. The upconverted PL spectra was calculated in the same fashion as described above except the polariton state populations below 2.45 eV in energy were not included in order to only show UP PL intensity. The integrated linecuts of the simulated PL were calculated by summing over the angular axis of the ARPL spectra. The Rabi splitting was set to 47 meV for all detunings.

Supporting Figures

a)

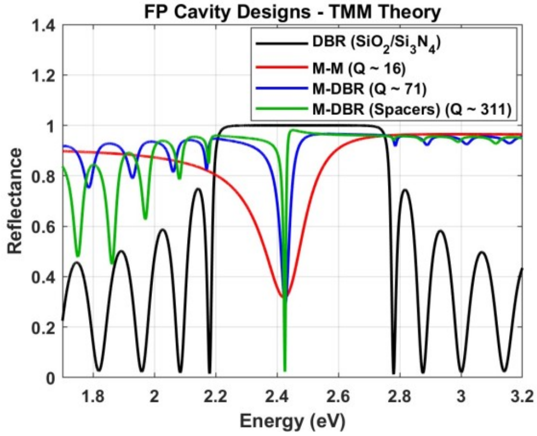

b)

Cavity Type

Layer	M-M	M-DBR	M-DBR (Spacers)
1	**Ag** *(40 nm)*	**Ag** *(40 nm)*	**Ag** *(40 nm)*
2	**NPL** *(77 nm)*	**NPL** *(105 nm)*	**SiO_2** *(200 nm)*
3	**Ag** *(120 nm)*	**SiO_2/Si_3N_4** *(15.5 pairs)*	**NPL** *(62 nm)*
4	-	-	**SiO_2** *(200 nm)*
5	-	-	**SiO_2/Si_3N_4** *(15.5 pairs)*

Fig. S1. Microcavity Design. a) Simulated reflectivity curves of various cavity geometries using the transfer matrix method for normal incidence across the 1.7 – 3.2 eV spectral window. **b)** Corresponding layers and their thickness for the different metal-metal and metal-dielectric cavities. For CdSe NPLs, an approximate average index value of 2.1 at the 2.42 eV HH transition was used and verified by measurements of film thickness on a Filmetrics F20 system. Adding SiO_2 spacers in a metal-dielectric cavity for a higher order $3\lambda/2$ cavity significantly increases the Q-factor while allowing for strong coupling.

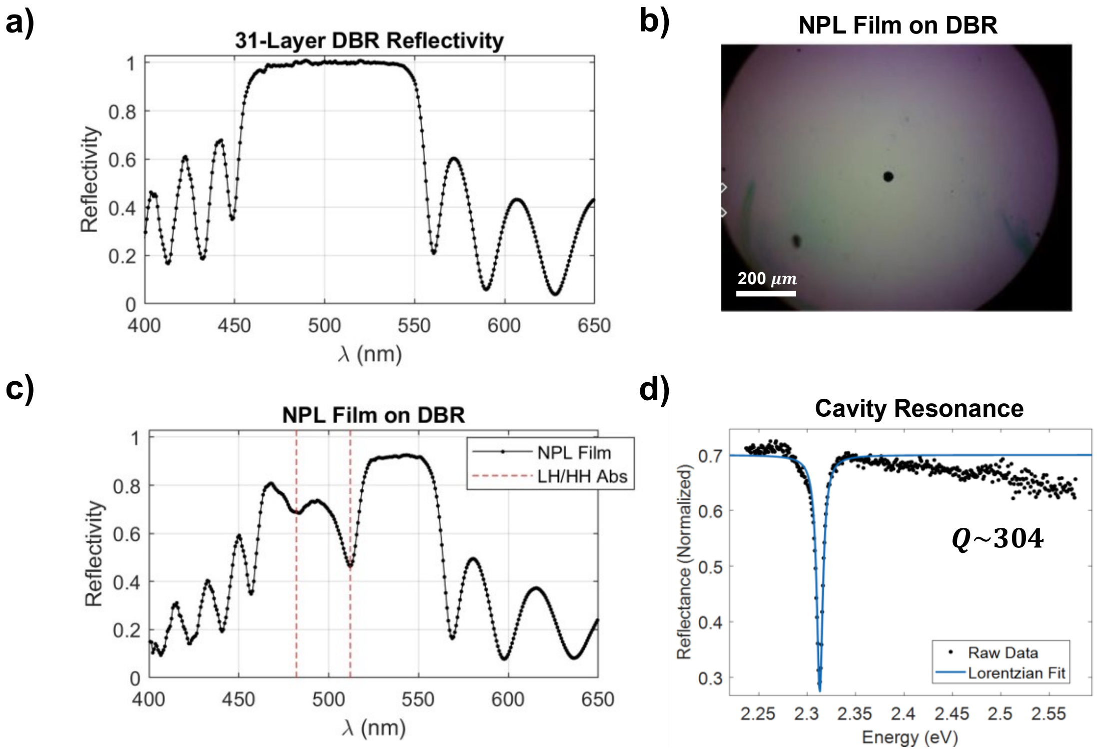

Fig. S2. Sample Characterization. a) Reflectivity of a 31-layer Si_3N_4/SiO_2 DBR stack on a silicon wafer fabricated via PE-CVD. **b)** Microscope image showing uniform NPL films deposited onto DBR mirrors via drop casting. **c)** Reflectivity of NPL film deposited onto the DBR showing clear LH/HH absorption transitions of the 4.5ML CdSe NPLs. **d)** Linecut (normal incidence) of an angle resolved reflectance spectrum from a highly detuned 536 nm cavity sample used to estimate Q-factor of 300 from a microcavity sample

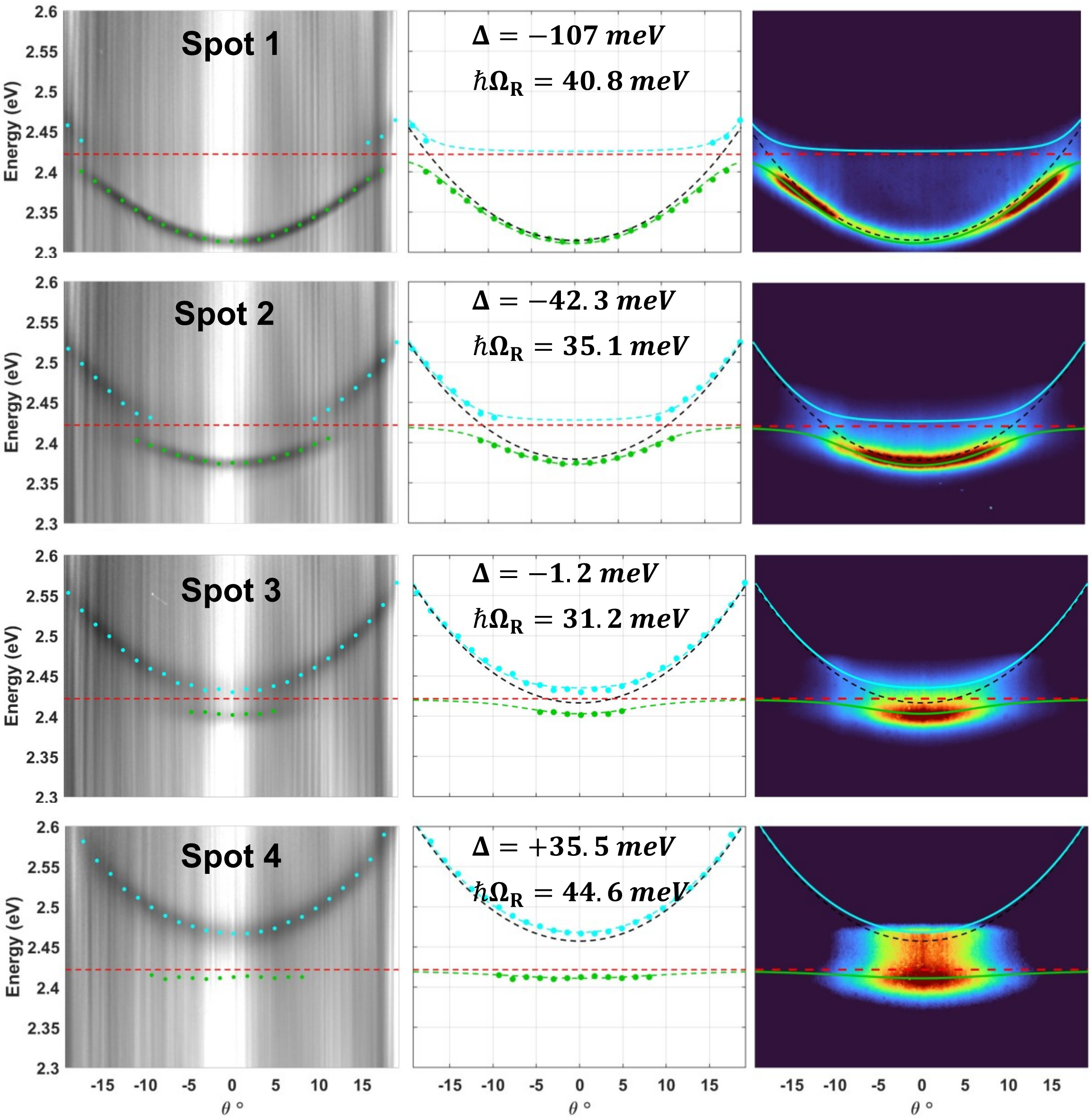

Fig. S3. Coupled harmonic oscillator model fittings (295 K). (Left) Raw fitted datapoints extracted from linecuts of reflectance images for 4 different sample positions with different cavity energy detuning (Δ). **(Center)** Coupled-harmonic oscillator model fitting for extracting Rabi-splitting energy ($\hbar\Omega_R$). **(Right)** Corresponding photoluminescence images with fit overlay. (Fig. 1d in main text corresponds to Spot 3).

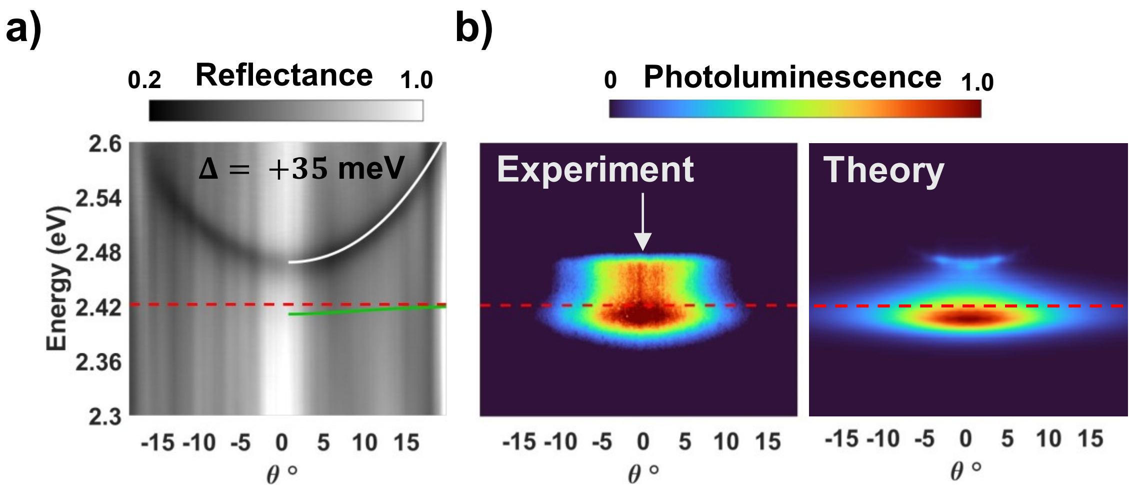

Fig. S4. Strong Coupling for Blue-Detuned Cavity (Spot 4, 295 K) – Experiment vs. Theory. a) Angle-resolved reflectance with fitted UPB (white), LPB (green), and exciton heavy-hole transition energy at 2.42 eV (dotted red) overlay for a sample with detuning energy $\Delta = +35\ meV$, Rabi-splitting energy $\hbar\Omega_{\mathrm{R}} = 44.6\ meV$, and $Q = 300$. **b)** Angle-resolved PL spectra indicating PL emission ranging from the LPB to the UPB (white arrow). Corresponding simulated quantum dynamical simulation showing agreement with measured PL emission concentrated at low angles for both the LPB and UPB.

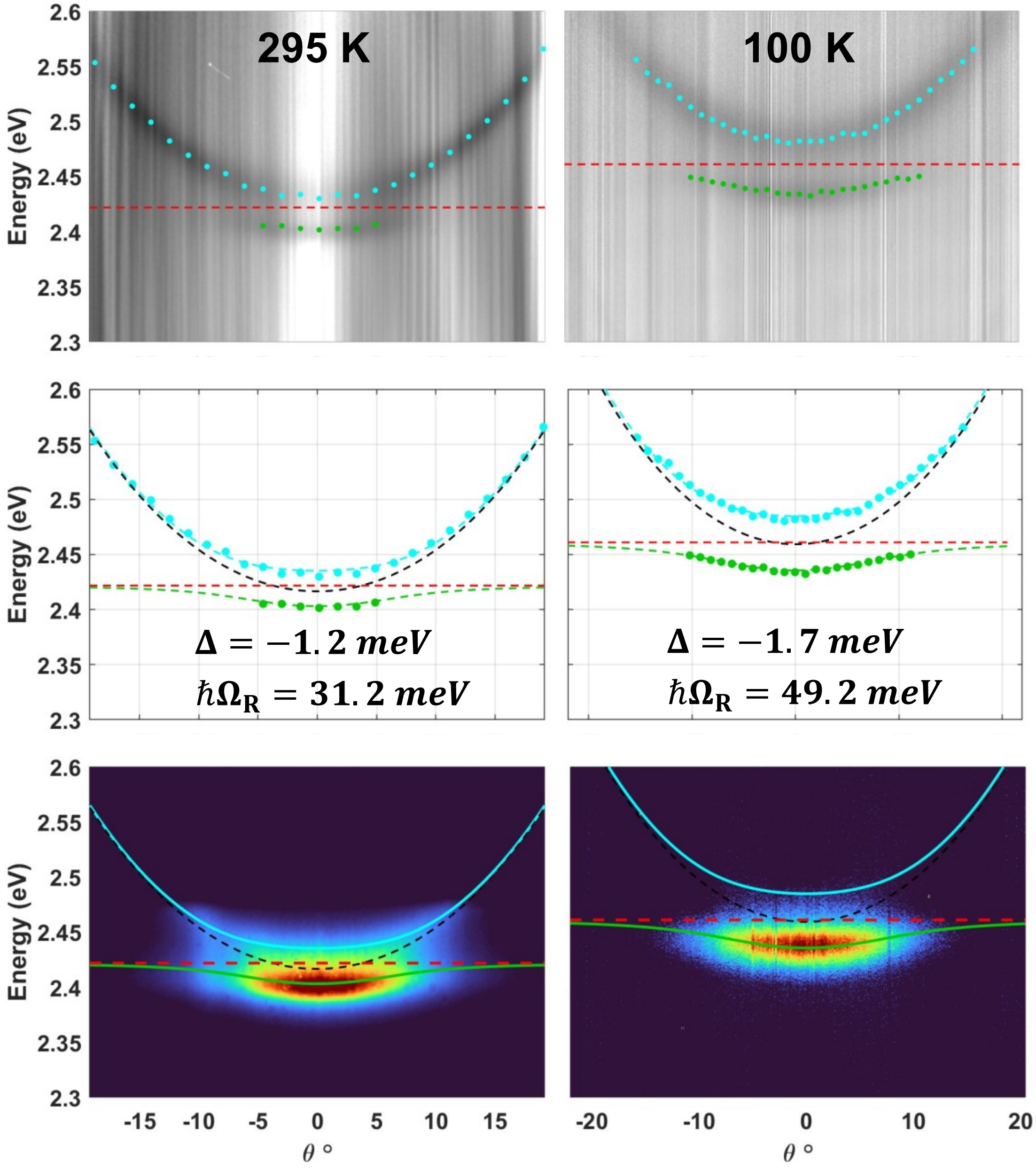

Fig. S5. 295 K vs 100 K Measurements. (Left) Raw fitted datapoints extracted from linecuts of reflectance images at 295 K **(Right)** and 100 K as shown in Fig. 2. PL emission from UPB branch is suppressed at 100 K under similar cavity detuning energy.

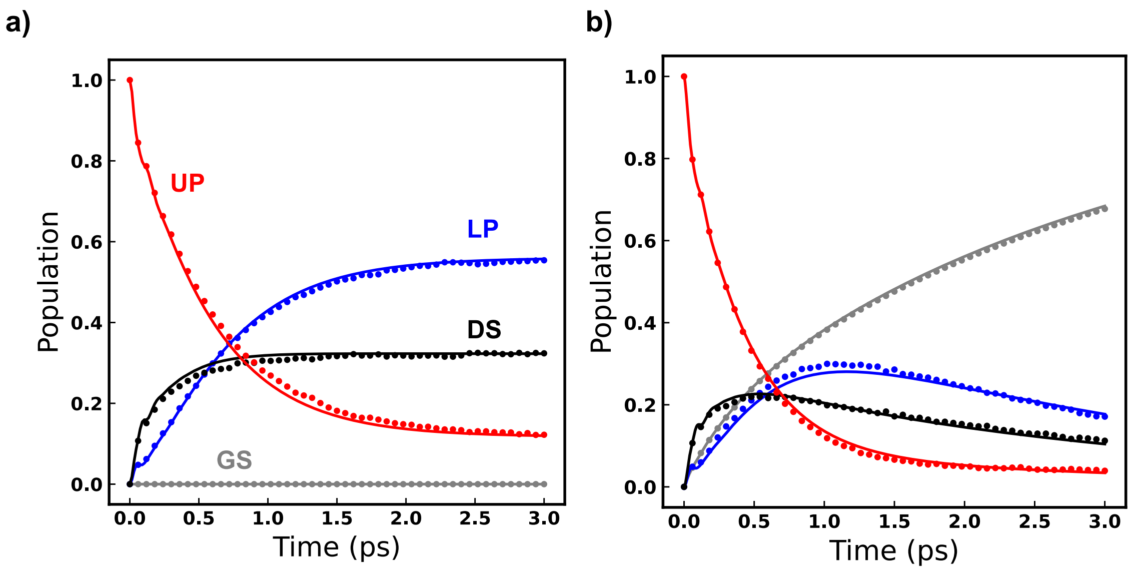

Fig. S6. Comparison of LMASH vs HEOM. a) Populations of HEOM (lines) and LMASH (dots) for a 6 molecule 3 mode GHTC model of the FP-NPL system with no cavity loss and parameters $\Delta = 0$ meV and $\hbar\Omega_R = 40$ meV. The populations are in the Tavis-Cummings eigenbasis. The sum of the 3 upper polariton state populations are in red, the sum of the 3 lower polariton state populations in blue, the sum of the 3 dark state populations in black, and the collective ground state population in gray. The system is initialized as a Franck-Condon excitation to the highest energy UP state. **b)** Same system as (a) but with cavity Q = 3000.

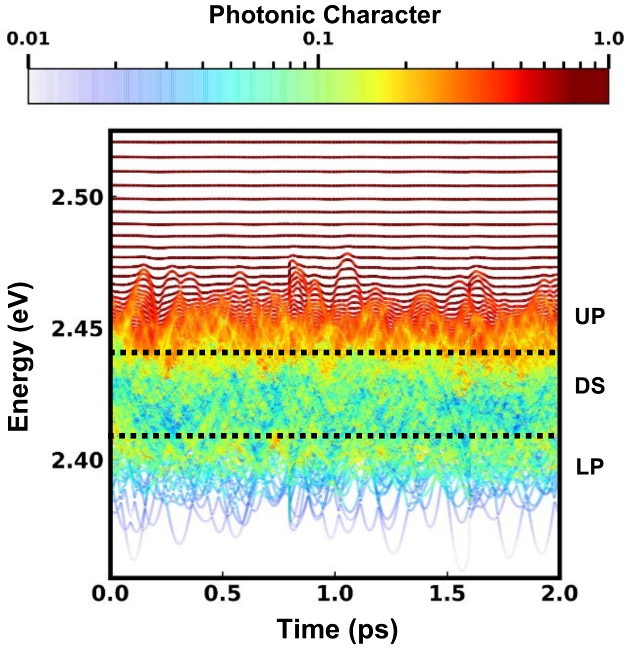

Fig. S7. Polariton State Trajectory. Polariton state energies through time colored by the total photonic character of each state for an arbitrary trajectory. Orange and redder regions correspond to greater photonic characters.

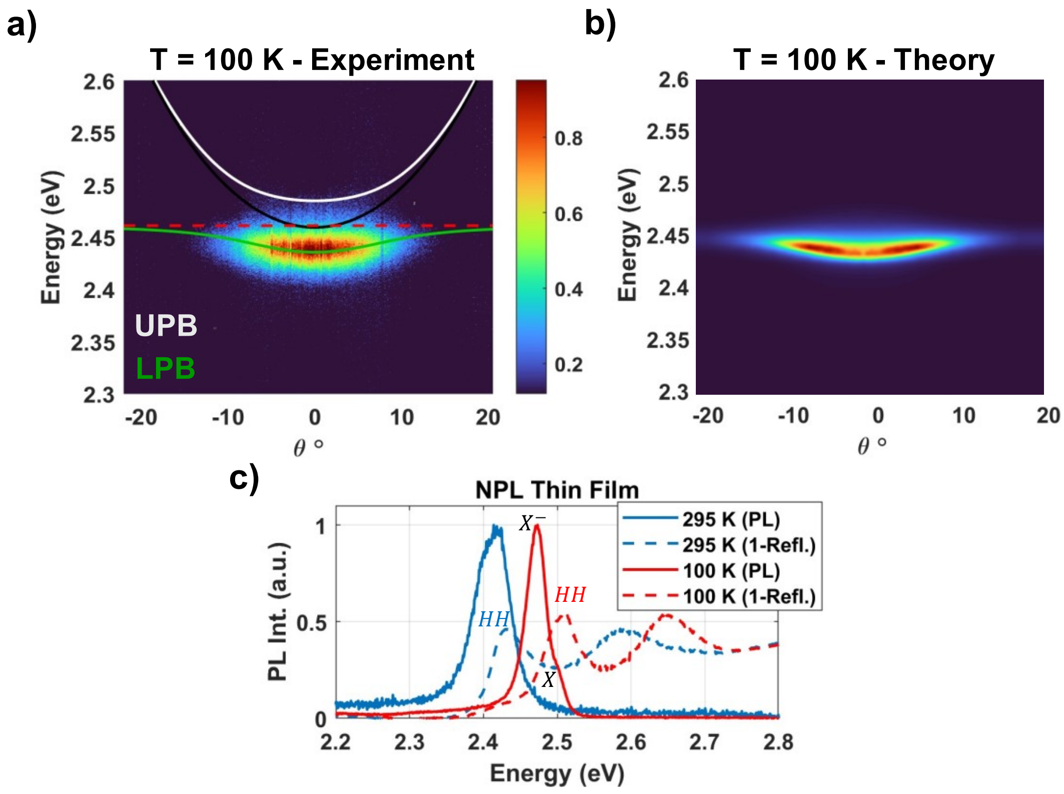

Fig. S8. Experiment vs Theory (100 K). a) ARR & ARPL plots at 100 K for a sample position corresponding to near resonant detuning (-1.7 meV) with fitted polariton branches. **b)** Theory simulations in excellent agreement with experiments showing dominant LP PL with minimal UP PL emission due to decreased phonon/dark states coupling compared to room temperature measurements. **c)** 4.5 ML CdSe NPL thin film PL (solid) and 1-Reflectance (dashed) taken at 295 K vs. 100 K showing exciton HH transition blue-shifts from 2.42 eV to 2.51 eV. Thin film PL is dominated by trion (X^-) emission at 2.473 eV which is redshifted by ~30 meV from the HH exciton (X) emission at 2.501 eV. Reflectance measurements only indicate cavity coupling to a HH transition at 2.46 eV due to small trion oscillator strength & lack of middle polariton branches. <u>Note:</u> Cavity HH redshift of ~50 meV from thin film measurements could be due to temperature dependent induced strain on the NPL film inside full cavity upon cooling or change in the electron-phonon coupling under strong coupling.

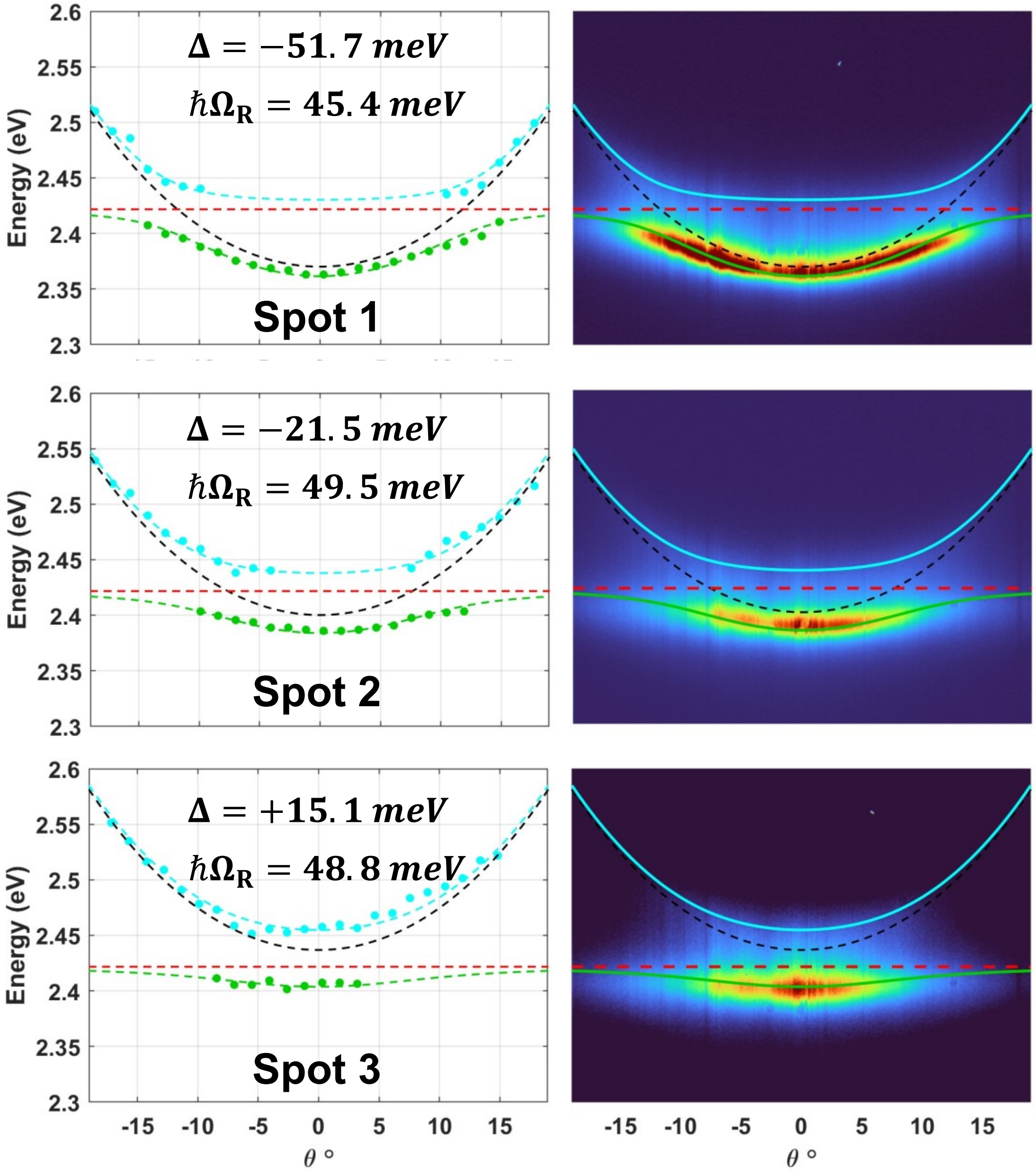

Fig. S9. Fitted Data for Upconversion Experiments (295 K). (Left) Coupled harmonic oscillator model fittings corresponding to three different positions & detuning (Δ) on the sample used for upconversion experiments in Fig. 3. **(Right)** Off-resonant excitation with CW 405 (3.1 eV) nm laser diode to obtain both UP & LP PL emission.

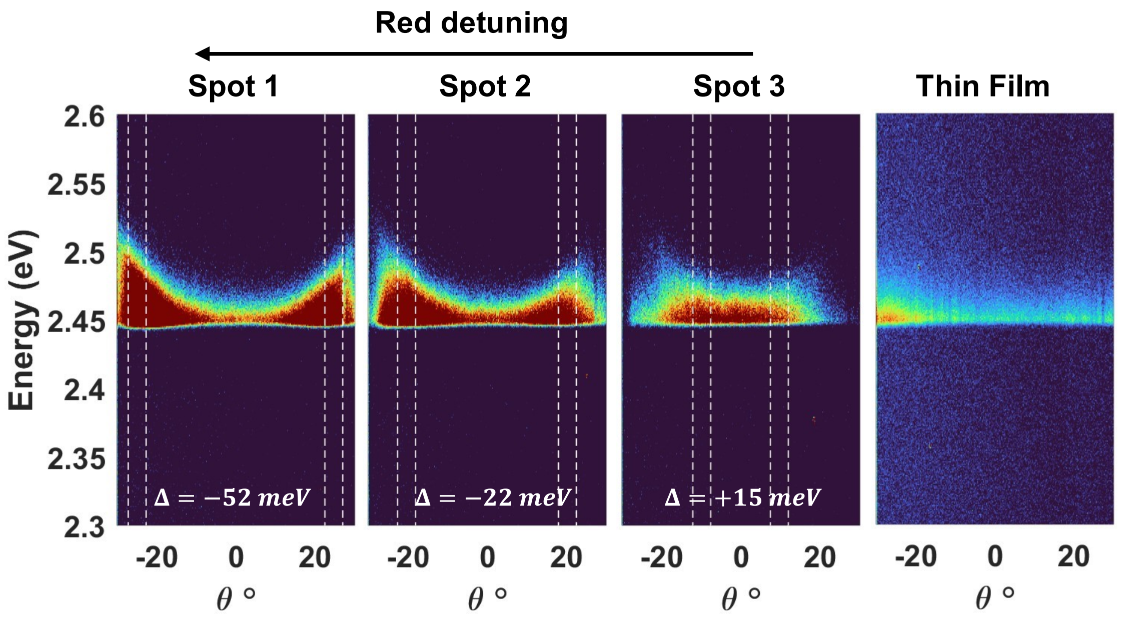

Fig. S10. Resonant LPB Excitation (295 K). Upconversion and PL emission from UPB corresponding to three positions on the microcavity sample with extracted fits in Fig. S9. Sample excited with pulsed white light continuum laser 515-545 nm (2.3-2.41 eV) & maximum UPB PL (2.39-2.5 eV) observed at angles corresponding to minimum detuning indicated by vertical dashed lines. Control measurement of a half cavity thin film sample showing weak exciton emission.

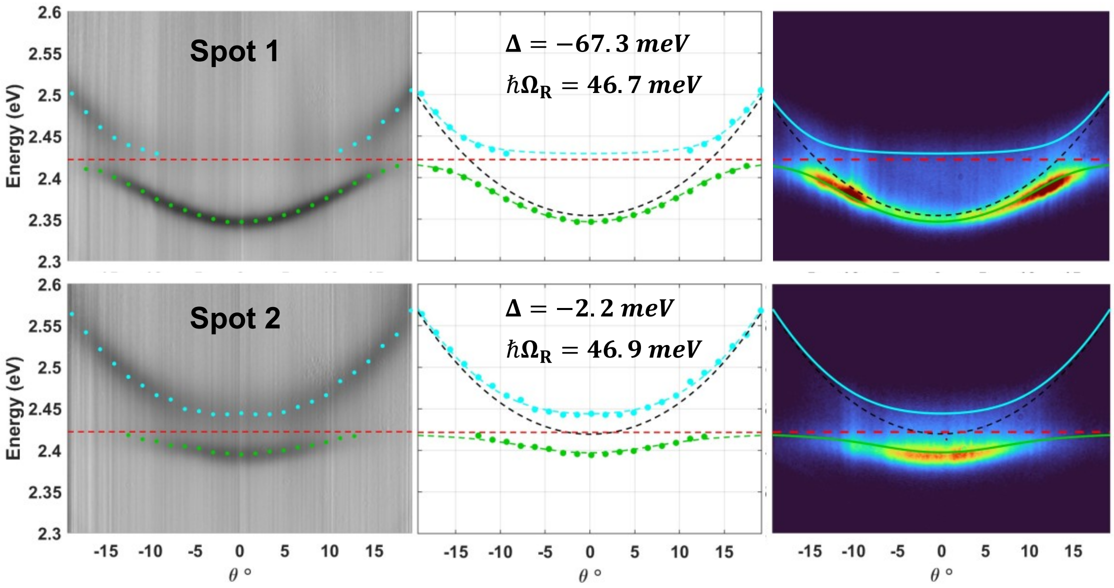

Fig. S11. ARR & ARPL Spectra for Lifetime Measurements (295 K). Comparison & fitting of angle resolved spectra for lifetime measurements corresponding to Fig. 4. **(Left)** Raw fitted datapoints extracted from linecuts of reflectance images for 2 different sample positions with different cavity energy detuning (Δ). **(Center)** Coupled-harmonic oscillator model fitting for extracting Rabi-splitting energy ($\hbar\Omega_R$). **(Right)** Corresponding photoluminescence images with fit overlay.

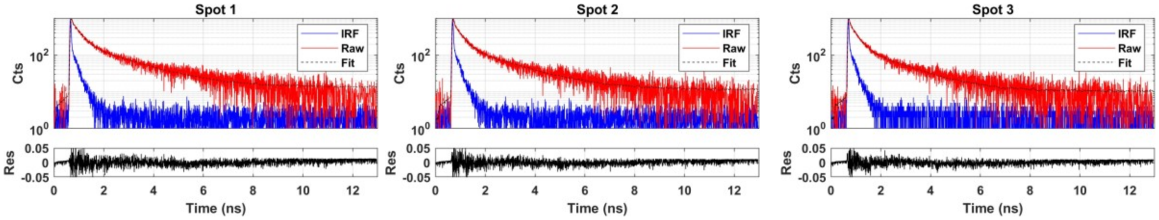

Fig. S12. Thin Film Lifetime Curves. Time correlated single photon counting (TCSPC) lifetime curves for three random positions on the control half cavity thin film sample measured at peak exciton emission of 513 nm (2.42 eV). Instrument response function (IRF) indicated by blue line; raw data measured at 4 ps time binning indicated by red line. Dotted black line corresponds to triple exponential deconvolution fitting along with residuals (bottom plot).

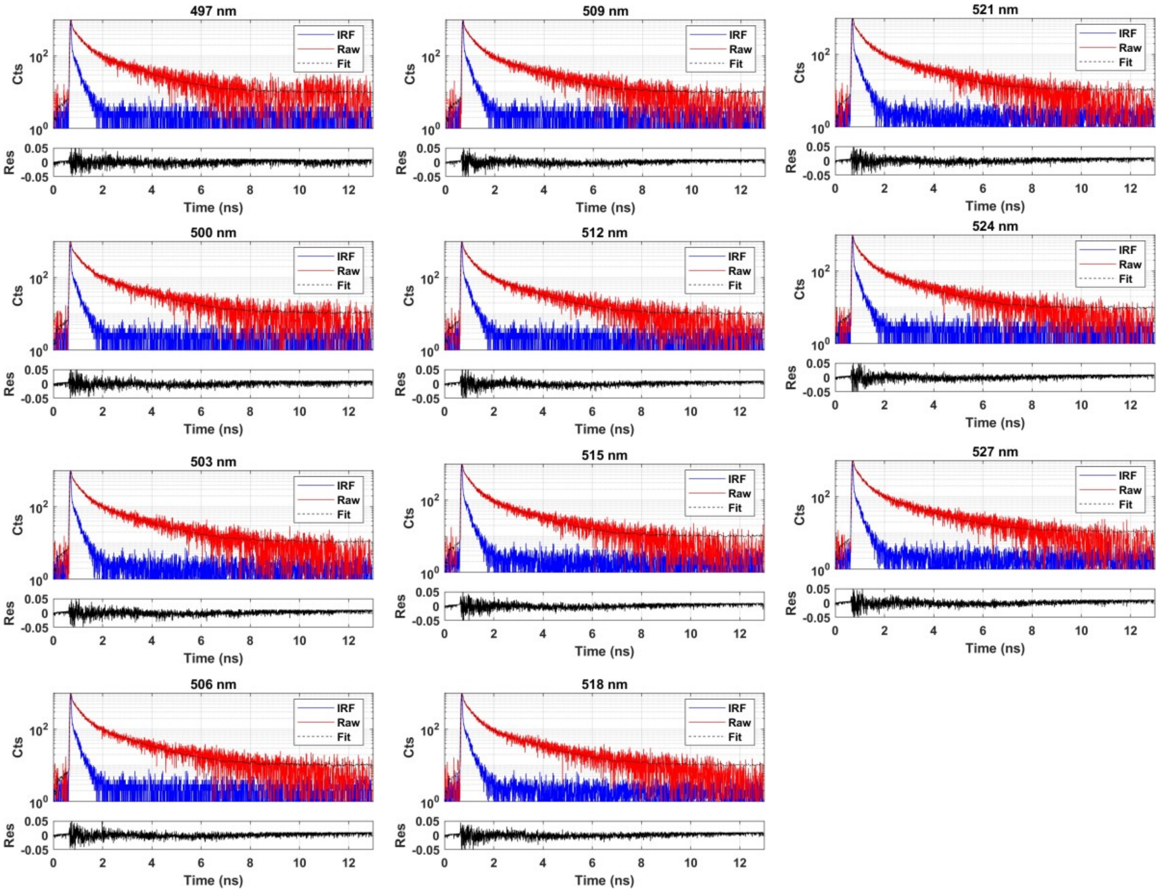

Fig. S13. Polariton Lifetime Curves. Time correlated single photon counting (TCSPC) lifetime curves for Spot 1 with Δ = -67 meV (Fig. S11) strong coupling sample measured across LPB & UPB every 3 nm using a grating spectrometer. Instrument response function (IRF) indicated by blue line; raw data measured at 4 ps time binning indicated by red line. Dotted black line corresponds to triple exponential deconvolution fitting along with residuals (bottom plot).

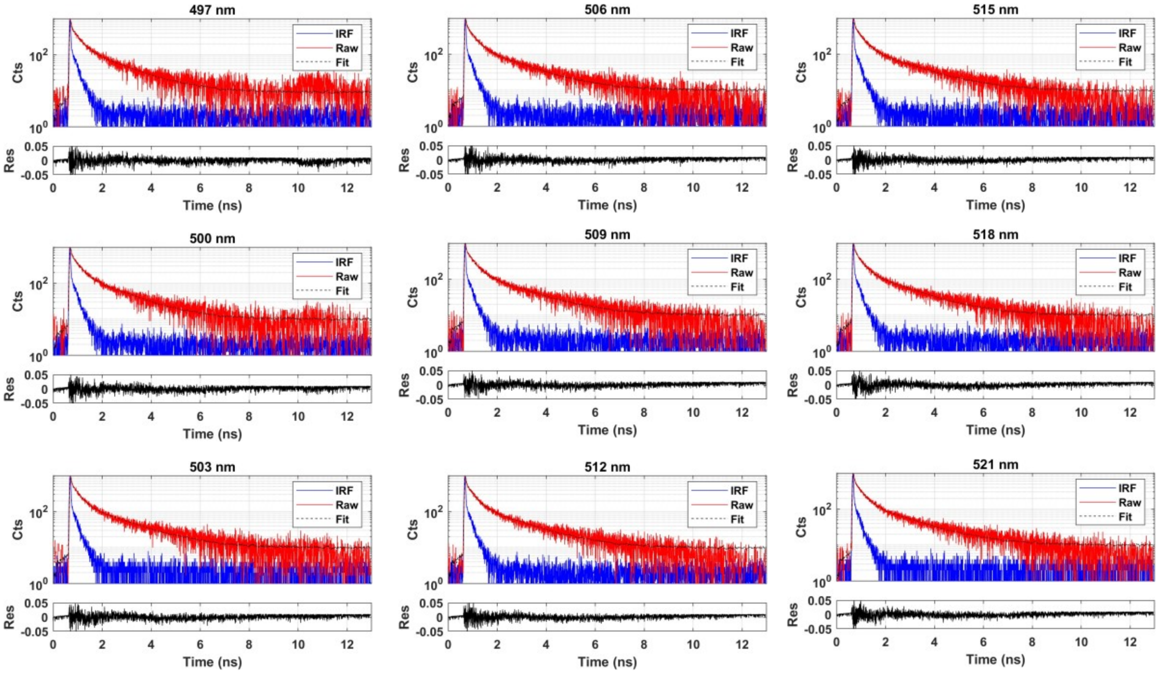

Fig. S14. Polariton Lifetime Curves. Time correlated single photon counting (TCSPC) lifetime curves for Spot 2 with Δ = -2 meV (Fig. 4 & Fig. S11) strong coupling sample measured across LPB & UPB every 3 nm using a grating spectrometer. Instrument response function (IRF) indicated by blue line; raw data measured at 4 ps time binning indicated by red line. Dotted black line corresponds to triple exponential deconvolution fitting along with residuals (bottom plot).

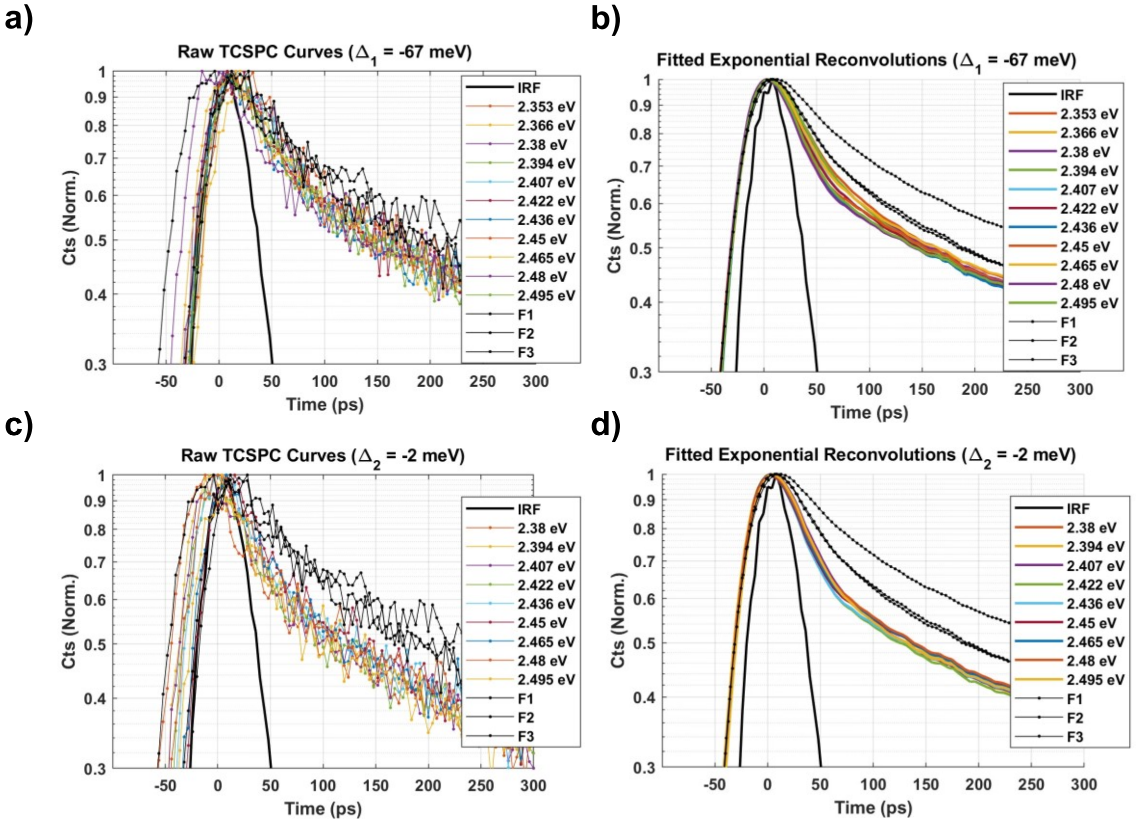

Fig. S15. Triple Exponential IRF Deconvolution Fits (Short Times). a) Raw TCSPC curves acquired for cavity detuning of Δ = -67 meV across all LP/UP energies along with three thin film (half-cavity) samples measured at the PL energy of 2.42 eV given by F1-F3. **b)** IRF deconvolved fits for short times showing small changes in lifetimes across polariton PL energies and deviations from thin film measurements. **c)** Raw and **d)** fitted curves for Δ = -2 meV cavity sample.

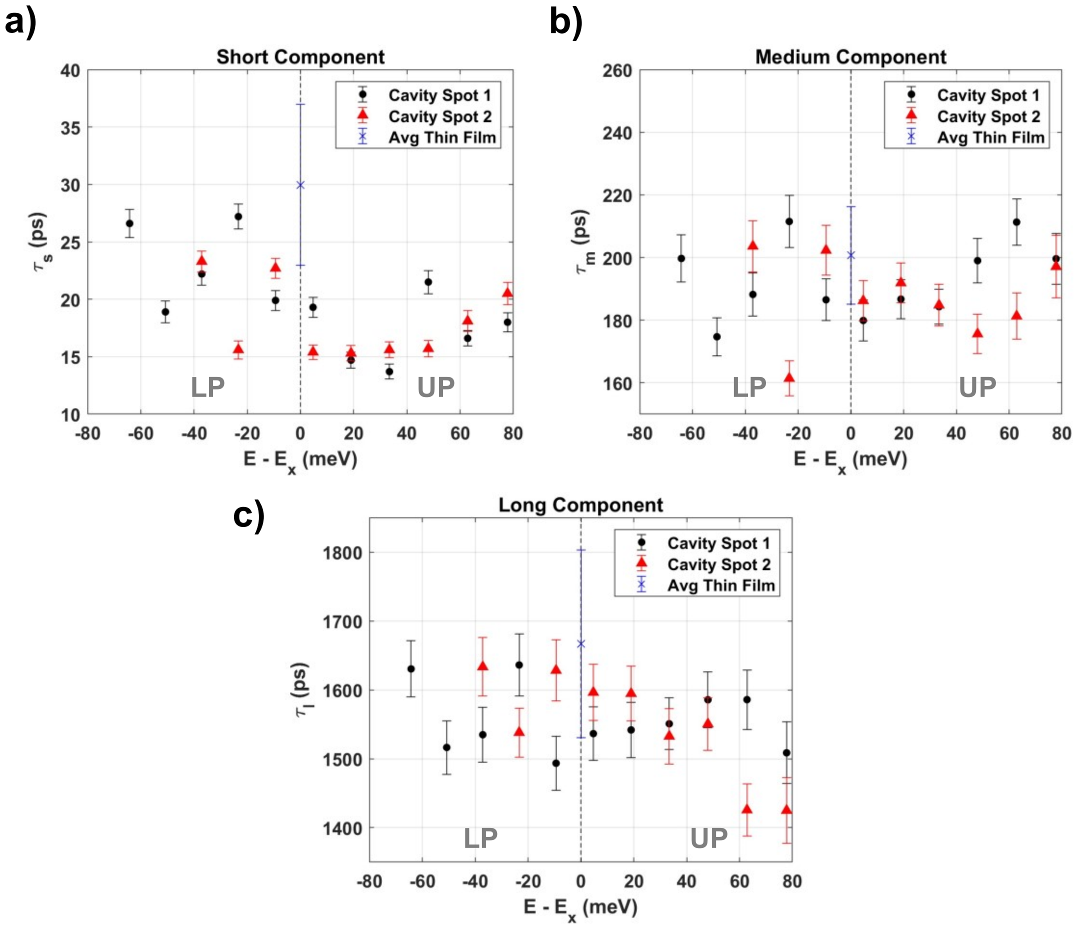

Fig. S16. Fitted Lifetime Components. Individual polariton lifetimes from the triple exponential deconvolution fits (see SI Tables S1-S3) as a function of difference in energies between bare exciton thin film PL emission at 2.42 eV and polariton PL emission (for Spot 1, Δ = -67 meV and Spot 2, Δ = -2 meV) – indicating that the **a)** shortest temporal component results in the largest difference between the cavity sample and bare thin films compared the **b)** medium and **c)** long components. Error bars for the cavity measurements correspond to 95% confidence intervals from fitting errors of individual components. Error bars on 'Avg Thin Film' correspond to the standard deviation across three thin film measurements.

Table S1. Individual time components and their normalized amplitude weights from triple exponential deconvolution fittings of a control half cavity thin film sample. Errors for three individual components correspond to 95% confidence intervals from fitting, while errors in the amplitude averaged lifetime ($\tau_{avg}^{amp} = A_1\tau_1 + A_2\tau_2 + A_3\tau_3$) correspond to propagation of the individual fitting errors.

Exciton Thin Film PL	$\boldsymbol{\tau_1(ps)\,,A_1}$	$\boldsymbol{\tau_2(ps), A_2}$	$\boldsymbol{\tau_3(ns), A_3}$	$\boldsymbol{\tau_{avg}^{amp}\ (ps)}$
**Spot 1**	37 ± 2.8 , 55.4%	213 ± 9.1 , 36.3%	1.74 ± 0.04 , 8.3%	242.6 ± 5.2
**Spot 2**	30 ± 1.7 , 67.4%	206 ± 8.6 , 26.9%	1.75 ± 0.05 , 5.6%	174.4 ± 3.9
**Spot 3**	23 ± 1.3 , 66.2%	183 ± 6.2 , 28.5%	1.51 ± 0.04 , 5.3%	146.8 ± 3.0

Table S2. Individual time components and their normalized amplitude weights from triple exponential deconvolution fittings of a strongly coupled cavity sample corresponding to Spot 1 in Fig. S11 with Δ = -67 meV. Errors for three individual components correspond to 95% confidence intervals from fitting, while errors in the amplitude averaged lifetimes correspond to propagation of the individual fitting errors.

$\lambda_{em}(nm)$	$\tau_1(ps)$, A_1	$\tau_2(ps), A_2$	$\tau_3(ns), A_3$	τ_{avg}^{amp} (ps)
**527**	27 ± 1.2 , 71.4%	200 ± 7.6 , 23.2%	1.63 ± 0.04 , 5.4%	153 ± 2.9
**524**	19 ± 0.9 , 73.4%	175 ± 6.1 , 21.9%	1.52 ± 0.04 , 4.7%	124 ± 2.4
**521**	22 ± 0.9 , 72.8%	188 ± 6.8 , 22.2%	1.53 ± 0.04 , 5.0%	134 ± 2.6
**518**	27 ± 1.1 , 73.6%	212 ± 8.2 , 21.4%	1.64 ± 0.04 , 5.0%	148 ± 3.0
**515**	20 ± 0.9 , 75.1%	187 ± 6.6 , 20.2%	1.49 ± 0.04 , 4.6%	122 ± 2.3
**512**	19 ± 0.9 , 74.9%	180 ± 6.5 , 20.5%	1.54 ± 0.04 , 4.6%	122 ± 2.3
**509**	15 ± 0.7 , 79.5%	187 ± 6.2 , 16.7%	1.54 ± 0.04 , 3.8%	101 ± 1.9
**506**	14 ± 0.7 , 79.1%	184 ± 5.5 , 17.0%	1.55 ± 0.04 , 3.8%	101 ± 1.8
**503**	21 ± 1.0 , 74.0%	200 ± 7.1 , 20.9%	1.59 ± 0.04 , 5.0%	137 ± 2.6
**500**	17 ± 0.7 , 79.9%	211 ± 7.3 , 16.0%	1.59 ± 0.04 , 4.1%	112 ± 2.2
**497**	18 ± 0.8 , 78.2%	200 ± 8.1 , 17.4%	1.51 ± 0.04 , 4.4%	116 ± 2.5

Table S3. Individual time components and their normalized amplitude weights from triple exponential deconvolution fittings of a strongly coupled cavity sample corresponding to Spot 2 in Figure 4 & Fig. S11 with Δ = -2 meV. Errors for three individual components correspond to 95% confidence intervals from fitting, while errors in the amplitude averaged lifetimes correspond to propagation of the individual fitting errors.

$\lambda_{em}(nm)$	$\tau_1(ps)$, A_1	$\tau_2(ps), A_2$	$\tau_3(ns), A_3$	τ_{avg}^{amp} (ps)
**521**	23 ± 0.9 , 77.2%	204 ± 8.2 , 18.2%	1.63 ± 0.04 , 4.7%	132 ± 2.6
**518**	16 ± 0.8 , 76.9%	161 ± 5.6 , 18.9%	1.54 ± 0.04 , 4.2%	107 ± 1.9
**515**	23 ± 0.9 , 77.2%	202 ± 8.0 , 18.3%	1.63 ± 0.04 , 4.5%	127 ± 2.6
**512**	15 ± 0.6 , 80.7%	186 ± 6.4 , 15.7%	1.60 ± 0.04 , 3.6%	100 ± 1.9
**509**	15 ± 0.7 , 80.7%	192 ± 6.4 , 15.5%	1.59 ± 0.04 , 3.8%	102 ± 1.9
**506**	16 ± 0.7 , 79.4%	185 ± 6.7 , 16.6%	1.53 ± 0.04 , 4.0%	105 ± 2.0
**503**	16 ± 0.7 , 78.6%	176 ± 6.3 , 17.2%	1.55 ± 0.04 , 4.2%	107 ± 2.0
**500**	18 ± 0.9 , 77.2%	181 ± 7.4 , 18.0%	1.43 ± 0.04 , 4.8%	115 ± 2.3
**497**	21 ± 1.0 , 78.2%	197 ± 10 , 17.0%	1.43 ± 0.05 , 4.8%	118 ± 2.9

Table S4. Parameters of the GHTC model for both off-resonant pumping (Figs. 1, 2, S4, S7, S8) and upconversion LP pumping (Fig. 3). The maximum photon mode frequency is $\omega_{\mathbf{k},\max}$, the number of discretized phonon bath modes per molecule is N_b, and the characteristic (peak) frequency of the spectral density is ω_{char}. The Rabi splitting, detuning, Q-factor, and temperature used vary based on the corresponding experimental sample.

**Pumping Type**	$\boldsymbol{N}$	$\boldsymbol{K}$	$\omega_{\mathbf{k},\max} - \omega_c$ **(meV)**	$\boldsymbol{N_b}$	$\boldsymbol{\lambda}_{\mathbf{HH}}$ **(meV)**	$\omega_{\mathbf{char}}$ **(meV)**	$\Gamma_{\mathbf{pump}}$ **(meV)**
**Off-Resonant Pumping**	160	40	150	200	5	4	$6/N$
**Upconversion LP Pumping**	160	80	200	200	5	4	$6/K$

SI References

(1) Max Born, E. W. *Principles of Optics*; Cambridge University Press, 1999. DOI: https://doi.org/10.1017/CBO9781139644181.
(2) Geiregat, P.; Rodá, C.; Tanghe, I.; Singh, S.; Di Giacomo, A.; Lebrun, D.; Grimaldi, G.; Maes, J.; Van Thourhout, D.; Moreels, I.; et al. Localization-limited exciton oscillator strength in colloidal CdSe nanoplatelets revealed by the optically induced stark effect. *Light: Science & Applications* **2021**, *10* (1), 112. DOI: 10.1038/s41377-021-00548-z.
(3) Diroll, B. T. Circularly Polarized Optical Stark Effect in CdSe Colloidal Quantum Wells. *Nano Letters* **2020**, *20* (11), 7889-7895. DOI: 10.1021/acs.nanolett.0c02409.
(4) Liu, J.; Guillemeney, L.; Choux, A.; Maître, A.; Abécassis, B.; Coolen, L. Fourier-Imaging of Single Self-Assembled CdSe Nanoplatelet Chains and Clusters Reveals out-of-Plane Dipole Contribution. *ACS Photonics* **2020**, *7* (10), 2825-2833. DOI: 10.1021/acsphotonics.0c01066.
(5) Qiu, L.; Mandal, A.; Morshed, O.; Meidenbauer, M. T.; Girten, W.; Huo, P.; Vamivakas, A. N.; Krauss, T. D. Molecular Polaritons Generated from Strong Coupling between CdSe Nanoplatelets and a Dielectric Optical Cavity. *The Journal of Physical Chemistry Letters* **2021**, *12* (20), 5030-5038. DOI: 10.1021/acs.jpclett.1c01104.
(6) Mandal, A.; Taylor, M. A. D.; Weight, B. M.; Koessler, E. R.; Li, X.; Huo, P. Theoretical Advances in Polariton Chemistry and Molecular Cavity Quantum Electrodynamics. *Chemical Reviews* **2023**, *123* (16), 9786-9879. DOI: 10.1021/acs.chemrev.2c00855.
(7) Tichauer, R. H.; Feist, J.; Groenhof, G. Multi-scale dynamics simulations of molecular polaritons: The effect of multiple cavity modes on polariton relaxation. *The Journal of Chemical Physics* **2021**, *154* (10), 104112. DOI: 10.1063/5.0037868.
(8) Arnardottir, K. B.; Moilanen, A. J.; Strashko, A.; Törmä, P.; Keeling, J. Multimode Organic Polariton Lasing. *Physical Review Letters* **2020**, *125* (23), 233603. DOI: 10.1103/PhysRevLett.125.233603.
(9) Berghuis, A. M.; Tichauer, R. H.; de Jong, L. M. A.; Sokolovskii, I.; Bai, P.; Ramezani, M.; Murai, S.; Groenhof, G.; Gómez Rivas, J. Controlling Exciton Propagation in Organic Crystals through Strong Coupling to Plasmonic Nanoparticle Arrays. *ACS Photonics* **2022**, *9* (7), 2263-2272. DOI: 10.1021/acsphotonics.2c00007.
(10) Jasrasaria, D.; Rabani, E. Circumventing the phonon bottleneck by multiphonon-mediated hot exciton cooling at the nanoscale. *npj Computational Materials* **2023**, *9* (1), 145. DOI: 10.1038/s41524-023-01102-8.
(11) Runeson, J. E.; Manolopoulos, D. E. A multi-state mapping approach to surface hopping. *The Journal of Chemical Physics* **2023**, *159* (9), 094115. DOI: 10.1063/5.0158147.
(12) Mannouch, J. R.; Richardson, J. O. A mapping approach to surface hopping. *The Journal of Chemical Physics* **2023**, *158* (10), 104111. DOI: 10.1063/5.0139734.
(13) Koessler, E. R.; Mandal, A.; Huo, P. Incorporating Lindblad decay dynamics into mixed quantum-classical simulations. *The Journal of Chemical Physics* **2022**, *157* (6), 064101. DOI: 10.1063/5.0099922.

(14) Johansson, J. R.; Nation, P. D.; Nori, F. QuTiP: An open-source Python framework for the dynamics of open quantum systems. *Computer Physics Communications* **2012**, *183* (8), 1760-1772. DOI: https://doi.org/10.1016/j.cpc.2012.02.021.
(15) Akimov, A. V. A Simple Phase Correction Makes a Big Difference in Nonadiabatic Molecular Dynamics. *The Journal of Physical Chemistry Letters* **2018**, *9* (20), 6096-6102. DOI: 10.1021/acs.jpclett.8b02826.
(16) Hu, D.; Mandal, A.; Weight, B. M.; Huo, P. Quasi-diabatic propagation scheme for simulating polariton chemistry. *The Journal of Chemical Physics* **2022**, *157* (19), 194109. DOI: 10.1063/5.0127118.
